# The first rodent behavioral study (1822) and the diffusion of human-bred albino rats and mice in the 19th century

**DOI:** 10.3389/fpsyg.2024.1532975

**Published:** 2025-02-03

**Authors:** Raffaele d’Isa

**Affiliations:** Institute of Experimental Neurology (INSPE), Division of Neuroscience (DNS), IRCCS San Raffaele Scientific Institute, Milan, Italy

**Keywords:** rats, mice, albino rats, albino mice, interspecies social bonding, history of behavioral sciences, history of comparative psychology

## Abstract

Rodents, in particular rats and mice, are currently the most widely employed animal models in psychology and behavioral neuroscience. Nevertheless, an interesting historical question is: when was the first rodent behavioral study performed and by whom? The current article presents the first rodent behavioral study in the history of science: a case of interspecies social bonding between a rat and a dog, observed in 1822 by the British chemist Samuel Moss (1794–1868) and subsequently described by the same in a scientific article in 1836. In the present article, after a biographical sketch of Samuel Moss, I examine in detail the notable case of interspecies bonding observed by Moss. This case is notable under several points of view. First, Moss’s rat was an albino, a variety which at that time was extremely rare. Moreover, at that time, in the Western world rats were mostly seen as pest animals or baits for rat-catching sports, and were not kept as pets. The color of the rat played a key role in its fate, being the reason for which it was originally brought to Moss and for which Moss decided to keep it under his care. Third, the relationship that arose between the rat and the dog is even more surprising if we consider that the dog was a trained rat-catcher. Importantly, this rat-dog bonding case, which showcased the tameness of Moss’s albino rat in both lay and scientific publications, represented the first popularization of the docility of albino rats. After having outlined Moss’s case, considering the importance of albino rats in our current society, both in scientific research (where the albino rat has become the prototype of the laboratory rat) and as pets, I provide an historical contextualization regarding albino rodents, starting from the 17th century, and I then trace the history of the post-Moss diffusion of human-bred albino rats and mice in the 19th century.

## 1 Introduction

Rodents, especially rats and mice, have become the most widely employed animal models in psychology and behavioral neuroscience (Logan, 2005; Maximino et al., 2015; Moulin et al., 2021; Shemesh and Chen, 2023; d'Isa and Gerlai, 2023). However, an interesting historical question is: when was the first rodent behavioral study performed and by whom? In the current article, I will present the first rodent behavioral study in the history of science: a case of interspecies social bonding between a rat and a dog, observed in 1822 by Samuel Moss and subsequently described by the same in a scientific journal in 1836. At first, I will outline a biographical sketch of Samuel Moss. Subsequently, I will describe in detail the notable case of interspecies bonding observed by Moss.

Interestingly, Moss’s rat was an albino, a variety which at that time was particularly rare. Moreover, at that time, in the Western world rats were mostly seen as pest animals or baits for rat-catching sports, and were not kept as pets. The color of the rat played a key role in its fate. Actually, the fact that the rat was white was the reason for which it was originally brought to Moss and for which Moss decided to keep it under his care. What happened next, the relationship that arose between the rat and the dog, was totally unexpected and is even more surprising if we consider that the dog was a trained rat-catcher. Importantly, this rat-dog bonding case, which showcased the tameness of Moss’s albino rat in both lay and scientific publications, represented the first popularization of the docility of albino rats.

After having outlined Moss’s case, considering the importance of albino rats in our current society, both in scientific research, where the albino rat has become the prototype of the laboratory rat (Logan, 1999, 2005; Kuramoto, 2012), and as pets (Patterson, 2006; Armentrout and Armentrout, 2013), I will provide an historical contextualization regarding albino rodents and I will trace the history of the post-Moss diffusion of human-bred albino rats and mice in the 19th century.

## 2 Samuel Moss: a short biography

**Figure 1:**
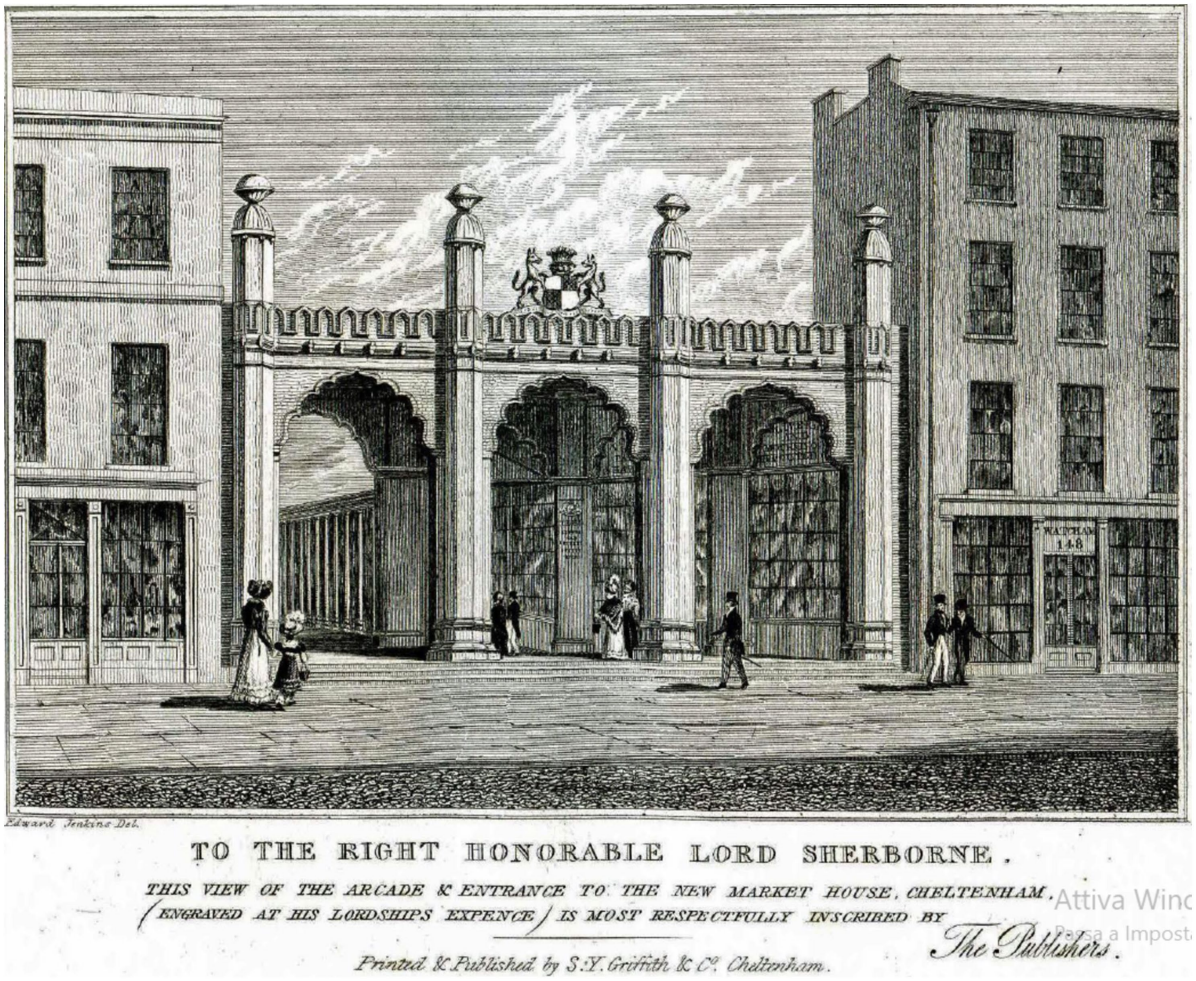
The pharmacy of Samuel Moss in High Street in Cheltenham. The figure shows the pharmacy of Samuel Moss in High Street, at the left of the monumental Mughal-gothic triple-arched entrance of the shopping Arcade, as represented by the British artist Edward Jenkins in an engraving included in the historical description of Cheltenham published in 1826 by Samuel Young Griffith (1796–1865), the proprietor of the *Cheltenham Chronicle* (Griffith, 1826). Originally numbered 150, Moss’s pharmacy was renumbered 149 in 1828, after that the adjoining building was replaced, in 1822–3, by the monumental entrance of the new shopping Arcade.

**Figure 2:**
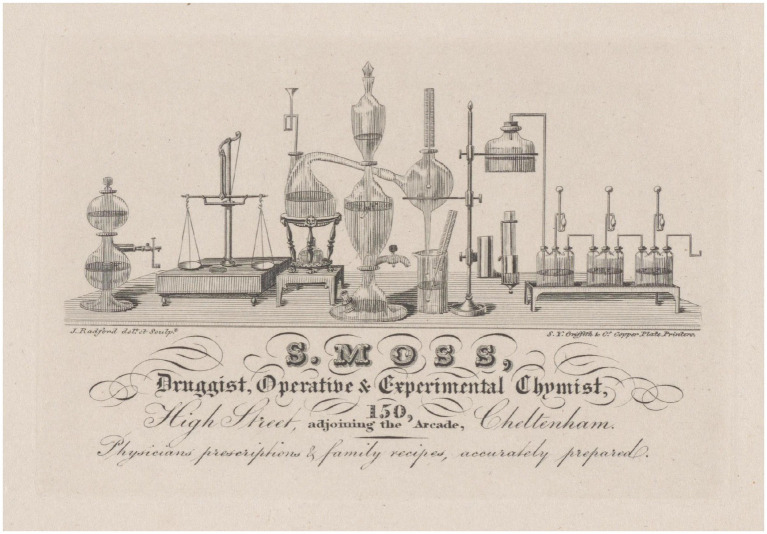
Trade card of Samuel Moss’s activity as chemist, pharmacist and electrotherapist. The figure shows an engraving used as trade card, currently kept at the Philadelphia Museum of Art and dated circa 1826, which advertised the services offered by Samuel Moss. Drawn and engraved by the Cheltenham artist and clock engineer Jonas Radford (active between 1826 and 1853). Printed by Samuel Young Griffith & Co. Copper Plate Printers. From: Philadelphia Museum of Art, The William H. Helfand Collection 1989, Item No. 1989-69-68. Courtesy of the Philadelphia Museum of Art.

Samuel Moss (1794–1868) was a British druggist, experimental chemist and pioneer of electrical medicine, who, in the 1820s and 1830s, was active in Cheltenham, a town in the county of Gloucestershire in England, United Kingdom. In Cheltenham, from 1820, Moss ran a pharmacy in High Street No.150 (Cheltenham Chronicle, 1820), renumbered No.149 in 1828 (Cheltenham Chronicle, 1828), after that the adjoining building was replaced, in 1822–3, by a monumental Mughal-gothic three-span arch that served as entrance to the new shopping Arcade and the nearby Market-House, both opened around 1823 (Dobney, 1823; Griffith, 1826; Landseer, 1828; Lewis, 1831). An engraving from 1826 shows the location of Moss’s pharmacy in High Street, to the left of the Arcade entrance (Figure 1). From the 1820s, Moss pioneered clinical electrotherapy, offering electrical stimulation treatments for several medical pathologies and employing electricity “as a restorative agent” in diseases (Cheltenham Chronicle, 1834). An engraving used as trade card, currently kept at the Philadelphia Museum of Art and dated circa 1826, advertises the services offered by Samuel Moss (Figure 2).

Moss’s scientific interests were wide. British physician and surgeon John Fosbroke (1787–1844), pioneer of clinical audiology and son of the historian and antiquarian Thomas Dudley Fosbroke (1770–1842), defined Moss as “a man of an ingenious and scientific mind” (Fosbroke and Fosbroke, 1826). Indeed, in addition to chemistry and electrical medicine, Moss also devoted himself to meteorology. Over the course of the 1820s and 1830s, he kept daily meteorological records, which he published weekly in the *Cheltenham Chronicle*. In 1832 Moss performed a meteorological experiment in which he elevated into the air, through a kite, pieces of beef meat, codfish and bread, in order to test the effect of the atmosphere on these samples and evaluate the quality of the air (Moss, 1832). In 1837 Moss was admitted as a member of the London Meteorological Society (Cheltenham Chronicle, 1837).

Moreover, Moss was also a member of the Cheltenham Zoological Society (Cheltenham Chronicle, 1836) and of the Cheltenham Scientific Institution (Cheltenham Chronicle, 1832). Subsequently, when the Cheltenham Scientific Institution was transformed into the Cheltenham Literary and Philosophical Institution in 1833, Moss became a member of the latter (Morning Post, 1833) and in 1838 he was part of the committee which received the task of developing a new section of the institution devoted to natural history (Cheltenham Looker-On, 1838).

After selling his drug store in 1839 (Cheltenham Looker-On, 1839), Moss moved from Cheltenham to Birkenhead, an English town on the western bank of the River Mersey, opposite to Liverpool, which is on the eastern bank. From 1840 Moss changed profession and worked primarily in the field of shipping, maritime trade and mariner management. The 1841 British National Census indicates that Moss lived in Birkenhead and had become a “mariner.” In Liverpool, Moss was active in several associations and charities, becoming Master of the Rooms for the Liverpool Shipmasters Association (Moss, 1841; Shipping and Mercantile Gazette, 1841), Secretary of the Liverpool Marine Society (Moss, 1866) and Secretary of the Liverpool Salvage Committee, which was devoted to the prevention and extinction of fires in the city (Liverpool Weekly Courier, 1868).

He died in Liverpool on 29 September 1868 (Liverpool Weekly Courier, 1868) and was buried in Cheltenham on 2 October 1868, as reported in the records of the St. Mary Parish in Cheltenham.

## 3 The case: an instance of interspecies social bonding between an albino rat and a terrier dog

This case of interspecies bonding, observed by Samuel Moss from 1822 to 1825, was first reported in a local newspaper, through just a few lines, in 1823 (Stamford Mercury, 1823), and subsequently described in full detail in 1836 by Moss himself in a scientific article (Moss, 1836) published in *The Magazine of Natural History, and Journal of Zoology, Botany, Minerology, Geology, and Meteorology*, a journal directed by the Scottish botanist John Claudius Loudon (1783–1843) and published in London by Longman, Orme, Brown, Green and Longmans (now Longman).

In his 1836 scientific article, Moss relays that, at the beginning of 1822, a white rat was caught in Cheltenham in the kennel or stables of Colonel William Berkeley (1786–1857), who at the time of Moss’s article had become Lord Segrave (Moss, 1836). In particular, according to Moss, the location where the white rat was found corresponds to where, a few years later, the “pleasure-ground” of the residential neighborhood Pittville was built.^1^ Due to the uncommon color of the rat, the animal “was considered a great curiosity.” Having received notice of this finding, Moss expressed interest in this type of rat and, shortly after, when a second white was caught in a trap, it was brought to Moss, who decided to keep it.

This white rat, found on 1 February 1822, was tamed by Moss and the notice of the tame white rat generated great interest, to the point that the local newspaper *Cheltenham Chronicle* devoted an article to it, in which the rat is described as “so tame and domesticated as to feed from the hand, run in and out of the cage playfully, and sit patiently and contentedly on the table, without manifesting the slightest inclination to escape” (Cheltenham Chronicle, 1822; reproduced in: Wood, 1864), The *Cheltenham Chronicle* article on the tame white rat had such an echo that it was rapidly reported also in the US press, as can be seen in the New York magazine *The Minerva* from 27 April 1822 (The Minerva, 1822). However, since the rat was harmed during the trapping, it survived only 3 weeks (Moss, 1836), so Moss kept this rat only for the first 3 weeks of February 1822.

In autumn 1822, another white rat was caught and brought to Moss (Moss, 1836). This is the rat that would become the subject of a most remarkable interspecies bonding case. This unusual case of bonding between a rat and a dog was perceived as so amazing that in 1823 it was reported, through a short dispatch, in the newspaper: “Mr S. Moss, chemist of Cheltenham, (formerly of Stamford), has in his possession a white rat, perfectly tame, and so familiarized as to come to him at his call; and it is a singular and amusing sight to see the animal at play with a dog, without the least dread, although the latter destroys common, or brown rats, whenever he can catch them” (Stamford Mercury, 1823).

In his 1836 scientific article, Moss provided an extensive and detailed description of the case. In autumn 1822, when Moss received it, the animal was “about three parts grown, and exceedingly savage.” Released in the sitting room, the rat immediately ran towards Moss “with great ferocity.” Moss housed the rat in a squirrel cage endowed with a turnabout wheel (a running wheel for physical exercise) and for the subsequent 2–3 days he provided food to the rat by hand. Initially, the rat snapped at the food and tried to bite Moss’s fingers through the wire mesh. The rat developed a sense of security for the box which was placed inside the home-cage as shelter and did not emerge from the box unless it was forced to do so. When Moss inserted his hand inside the box and tried to get the rat out, he was bitten severely and this happened again for the subsequent 2–3 times.

However, Moss reports that, since the rat was always treated with kindness notwithstanding its first attacks, it “soon ceased to exhibit any sign of anger” and, from then on, when Moss lifted the lid of the box to see it, the rat just laid passively without ever launching any other attack. In the following days, Moss started releasing the rat from the cage to let it roam about the room, so that it could familiarize with the environment and with him. In order to facilitate the process of familiarization, during these sessions Moss, who was at the table occupied in reading or writing, carefully avoided showing the rat that it was being watched and did not interfere with it. The rat moved freely around the room and approached Moss crossing his feet under the table to collect and eat the fallen crumbles, which were actually purposely dropped by Moss for it. In about 2 weeks, the rat became very confident towards Moss, to the point that, when called, it would approach Moss and eat sugar or bread from his hand.

In that period, Moss had a dog, a small female white terrier named Flora, who happened to be a dog trained in rat-catching and an extremely proficient rat-killer. When the rat was first brought in the room inside the cage, Flora appeared “very anxious to get at him.” However, the first time that Moss took the rat in his hand, he called Flora and familiarized her with it. After that, Flora never showed “the slightest wish to assail the rat.” Apparently, the rat, that Moss named Scugg, was accepted by the dog as a member of the house’s social group. In a short time, the two animals became very attached. Moreover, Flora even showed protective behaviors towards the rat and the rat in turn appeared to seek her protection. Indeed, when any stranger came into the room, “the rat put himself under her protection, by going into a corner of the room, while Flora stood sentry, growling and showing her teeth most furiously, until satisfied that no injury was meditated against her favourite.”

At the back of his house, Moss had a garden surrounded by high walls. There, Moss frequently kept the rat and the dog free so that they could run and play together. The two appeared to play a sort of playful chasing game, “playing at hide and seek among the flowers.” However, when Moss blew his whistle, they immediately stopped playing and they both ran to Moss to pay their respects to him.

When Moss was at the table for meals, Scugg used to run up Moss’s leg and get on the table, carrying away the pieces of food that he^2^ could find, such as pastry or cheese, and bringing them on the floor, where he nibbled them a little and then left them for Flora. When Flora was particularly hungry and eager to have the first bite, Scugg kept her back “by striking her on the nose with his fore paw, which Flora never resented, but would sit quietly looking on, until permitted to take her share.” As for drinking, the rat and the dog lapped milk from the same saucer.

In the resting time, Scugg and Flora stayed together in front of the fire, with Scugg sleeping between Flora’s legs. Scugg also developed a strong attachment towards his human caregiver. At home, Scugg remained for hours in physical contact with Moss, lying inside his waistcoat. When he was taken out by Moss, he stayed in Moss’s pocket.

Some acquaintances of Moss suggested that the rat could have been protected from the aggressivity of the rat-killing dog by its white color. However, Moss relays an event that confutes this hypothesis. In November 1824, another white rat was caught and brought to Moss (this was the third albino rat that Moss received). This white rat was carried to Moss’s house inside a trap and arrived in the evening, when Scugg was free and was playing with Flora around the room. Moss opened the trap and shook out the newcomer towards them. Suddenly, the two rats started to run all about and Flora ran after them. Almost immediately, one of the rats was caught by the dog and killed, with great consternation of Moss, who in that confusion could not even distinguish which of the two was caught. Then, with great relief and joy, Moss saw a rat that he clearly identified as Scugg running to the corner and Flora setting herself in front of Scugg to defend him, position which she kept for all the time that the dead rat and the man who brought it remained in the room.

In February 1825, Moss got married and he separated from Scugg. Unfortunately, Scugg lived only 3 or 4 weeks after this separation, dying at the end of March 1825.^3^ The reason of Scugg’s death remains unclear. Moss declares: “Whether he grieved at parting, or whether he was not kept sufficiently clean, or was not allowed sufficient liberty, I cannot tell.” In any case, during this period Moss saw Scugg several times. The last time Moss encountered Scugg, he noticed that it was quite difficult to collect the rat from his chest and put him back inside the cage, as if he refused leaving Moss. Once back in the cage, Scugg went in a corner of his box and curled up. The next morning, he was found dead in the same position. Since when Scugg was brought to Moss (around November 1822) he was approximately 1.5 months old (“about three parts grown”), at his death (late March 1825) he was about 30 months old. Considering that the life expectancy of wild rats is very short and that over 90% die within 1 year of age (Feng and Himsworth, 2014), Scugg certainly lived far beyond the average of his wild counterparts.

## 4 Historical contextualization

From an historical point of view, the case reported by Moss is notable for both the animal subject and the topic. Indeed, the animal subject, an albino rat, was a rarity at that time. While now albino rats are common in human societies both as pets and in scientific research, in 1822, when Moss first observed the case, albino rats in Europe were not commercially available, were not kept as pets and were not employed in scientific research. In order to provide an historical contextualization and allow an understanding of the place of Moss’s case in the chronology of the interactions between albino rodents and humans, in the following sections I will outline a history of albino rats and albino mice in human society from the 17th century to the early 20th century, a time frame in which these animals were employed in mainly four contexts: baits in rat-catching sport competitions (section 4.1), as pets (section 4.2), in public entertainment animal shows (4.3) and in scientific research (section 4.4).

The co-protagonist of Moss’s case is also remarkable, as Flora was not simply a dog, but a terrier trained in rat-killing. In the England of the 1820s, rat-baiting competitions were particularly popular and terriers were the main dog breed used in this sport. A history of rat-catching sports in the 19th century is described in section 4.1.

Finally, the topic of Moss’s case, interspecies social bonding, is particularly noteworthy. Indeed, the European and American newspaper articles reporting about Moss’s first tame white rat in 1822 (Cheltenham Chronicle, 1822; The Minerva, 1822) were the first popularization of the docility of albino rats, while the 1823 article (Stamford Mercury, 1823) was the first popularization of an amicable social relationship between a rodent and a non-rodent non-human animal. At that time, this interspecies relationship was received with great surprise and with enthusiastic admiration. Interestingly, over the course of the 19th century, the interaction of albino rodents with other animals became a popular attraction among animal shows, which became known as the “Happy Family” show. The history of Happy Family exhibitions is described in section 4.3.

### 4.1 Rat-baiting

One of the most singular aspects of the dog-rat interspecies bonding described by Moss is that the dog was not simply a domestic dog, but a skilled rat-killing terrier. Indeed, at that time, many dogs, especially terriers (such as bull and terrier, bull terrier and Jack Russell terrier), were trained to catch and kill rats, for pest control or for blood-sport shows featuring rat-baiting. Rat-baiting shows were public exhibitions in which a great number of rats (often 100) were released in an enclosed space together with a dog and the time required for the dog to kill all the rats was measured with a stopwatch. The people in the audience could bet on the outcome, both in the case that the dog was competing against time or against the performance of a second dog (in which case two consecutive challenges were performed for the two dogs and the dog with the best result was declared the winner).

**Figure 3:**
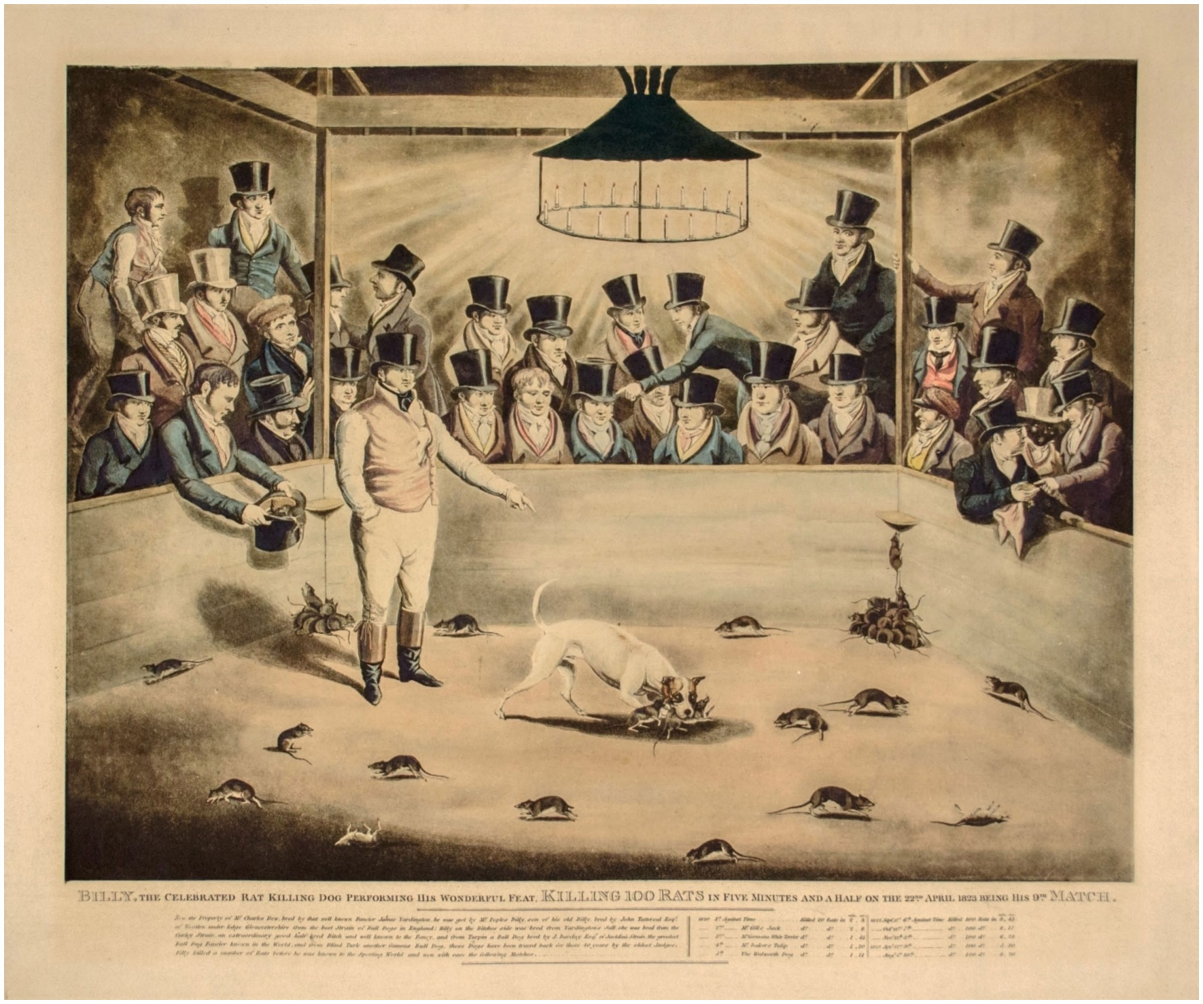
Rat-catching sport contest in 1823. A hand-colored engraving dated ca. 1823 represents the famous dog Billy in action in a rat-baiting show held on 22 April 1823, when Billy achieved his record, killing 100 rats in 5 min and 30 s (3.3 s per rat). Engraving title: “Billy, the Celebrated Rat Killing Dog Performing His Wonderful Feat. Killing 100 Rats in Five Minutes and a Half on the 22nd April 1823 Being His 97th Match.” Courtesy of the George Glazer Gallery of New York City (www.georgeglazer.com).

For instance, in the same year in which Moss observed the case of Scugg and Flora (1822), the famous dog Billy, a terrier and bull mixed breed, was exhibited at the Cockpitt of Westminster in London as a spectacular example of efficiency in rat-killing (The Sporting Magazine, 1823). On 24 October 1822, “the lovers of rat-killing enjoyed a feast of delight in a prodigious raticide,” with Billy challenged to kill 100 rats in 12 min. The dog completed the task in 7 min and 17 s (4.4 s per rat). In a following show held on 13 November 1822, Billy performed even better, killing 100 rats in 6 min and 25 s (3.9 s per rat). As reported in a hand-colored engraving dated ca. 1823 (Figure 3), on 22 April 1823 Billy achieved his record, killing 100 rats in 5 min and 30 s (3.3 s per rat). Indeed, British terriers were so proficient in rat-catching that they were subsequently used in the United States to create a new canine breed specialized in this task, the rat terrier, which became a common farm dog in the early 20th century (Mehus-Roe, 2011).

Rat-baiting continued to be very popular in the following decades. In 1851, the British journalist and social researcher Henry Mayhew (1812–1887), who investigated and documented with a series of interviews the life of London’s working class, relayed that there were 40 places in London hosting this blood-sport, although some were active only in the holiday times (Mayhew, 1851).

In the 1840s and 1850s, one of the main rat-baiting pits of London was the Blue Anchor Tavern at 102 Bunhill Row, owned by John James Shaw (also known as James Shaw and Jimmy Shaw), a former pugilist (Dickens, 1850; Rodwell, 1850; Mayhew, 1851, 1861). Mayhew described Shaw as the “proprietor of one of the largest sporting public houses in London, who is celebrated for the rat-matches which come off weekly at his establishment” (Mayhew, 1861). Interviewed by Mayhew, Shaw disclosed that he employed “from 300 to 500 rats a-week” for his rat-baiting matches, which means “a yearly purchase of 26,000 live rats” (Mayhew, 1861). At a certain point, Shaw arrived to host in his establishment the extraordinary number of 2000 rats.

Rat-baiting gradually lost popularity in the last decades of the 19th century, partly due to the complaints of the animal welfare movement, and was legally forbidden by the Protection of Animals Act of 1911, which, among the offences of animal cruelty, enlisted the cases in which a person “shall cause, procure, or assist at the fighting or baiting of any animal; or shall keep, use, manage, or act or assist in the management of, any premises or place for the purpose” (UK Protection of Animals Act, 1911).

### 4.2 Early breeding of albino rodents as pets

Albino mice and rats are currently common in human society, both as pets and in scientific research. On the other hand, in nature albino mice and rats are extremely rare. Hence, we can understand that these albinos derive from artificial selection through selective breeding by humans. The origin of these breeds is still unclear. However, it appears that the original reason for which these animals were collected and bred by humans was to keep them as fancy pets. There is documentation that, both in Far East and in Europe, albino rodents were kept as pets at least from the 17th century. However, it is in the 18th century that the habit of keeping albino rodent pets became more consolidated.

In Japan, albino rodents, especially rats, became particularly popular pets in the Edo period. Indeed, by the end of the 18th century, the habit of keeping pet rats became so widespread that the first two guidebooks on raising and keeping domestic rats were published: *Yoso-tama-no-kakehashi* (1775) and *Chinganso-date-gusa* (1787).

The world’s first guidebook on rat breeding, *Yoso-tama-no-kakehashi*, was written by a rat breeder from Osaka, known as the owner of the “Shumpo-do.” In this book, the author describes several artificially-selected varieties of rats (“nezumi”) and provides practical advice for the mating, pup raising, housing, feeding, disease treatment, taming and trading of the rats (Kuramoto, 2011). Illustrations are included to make the content of the book more easily understandable. Among the mentioned rat strains, the most important is the albino rat, which included two subtypes: the “red-eyed white” and the “black-eyed white.” From the albino variety, through further crossings, special varieties with unusual piebald coat patterns were created, such as the black and white spotted rat, the “bear rat” (which had a black coat with a crescent moon-shaped white ring on the breast), the “deer-spotted rat” (which had a mottled coat with many white spots like the dear) and the “cracked rat” (which had a unilateral pigmentation of the fur of the head). Interestingly, the author reports that in that time in Osaka there were five shops that were buying and selling pet rats, four in Shinsai-bashi Street and one in Sanoya-bashi Street.

The second guidebook, the *Chinganso-date-gusa*, was published by Jyo-En-Shi and contains similar practical information for the breeding and keeping of these rodents. Additionally, it relays a story about the origin of rat breeding in Japan, stating that it was carried out as early as 1654, when the Chinese Buddhist monk Ingen-zenji (1593–1673) moved to the Kyoto prefecture bringing with him a black-eyed albino rat from China, which he kept as pet and was admired by the visitors of the Obakusan-Manpukuji temple that Ingen-zenji had founded near Kyoto, who started wanting a rat of the same type (Nishikawa and Aiura, 2015; Hedrich, 2020).

In Europe, there are records of pet albino rodents from the late 17th century. From the correspondence of the French writer Marie de Rabutin-Chantal (1626–1696), better known as Madame de Sévigné, we learn that in 1672 in Paris the philosopher François de La Rochefoucauld (1613–1680) had a pet white mouse, which he considered an example of beauty and he showcased to his friends. After having seen the animal, Marie de Sévigné, in a letter dated 5 February 1672 and addressed to her daughter, the Countess of Grignan Françoise-Marguerite de Sévigné (1646–1705), relayed: “Monsieur de la Rochefoucauld tells you that he has a white mouse which is as beautiful as you; it’s the prettiest beast we have ever seen; it’s in a cage” (de Rabutin-Chantal, 1818).^4^

In 1757, the French physicians and naturalists Louis Daniel Arnault de Nobleville (1701–1778) and François Salerne (1706–1760) published the sixth volume of their zoological encyclopedia. Interestingly, in this volume the authors state that mice “are easily tamed; and once they are tamed, they do not seek to escape” (de Nobleville and Salerne, 1757).^5^ They relate that at their time some people enjoyed keeping pet mice in special small cages with a spinning wheel and describe: “It is a pleasure to see how quickly these animals turn the wheel: when they get tired, they return to the house to rest. You can accustom a rat to the same maneuver” (de Nobleville and Salerne, 1757). The authors then mention having seen a man that was keeping as pet a white mouse: “We saw a mouse white as snow, which his master carried with him everywhere in a small box; it ate bread or other food with familiarity from his hand, and after walking around the desk or the table, it returned of its own will inside its box” (de Nobleville and Salerne, 1757).

John Carteret Pilkington (1730–1763), an Irish singer and writer who left vivid memoirs of his life, in 1761, when living in London, was eager to purchase a cage so that he could deliver, as pet animals, two white mice, that had just arrived from the East Indies, to the Duchess of Marlborough Elizabeth Spencer (c. 1713–1761), who had a long-standing passion for white mice (The Westminster Magazine, 1777; Carey, 1792; Addison, 1797; Grin, 1800; Goldsmith et al., 1801; Clarke, 2016). This episode not only allows us to know that in 1761 England albino mice were already kept as pets, but also sheds light on the provenance of these animals (Far East). However, although these pets are described as a rarity, it is likely that this was not the first importation of white mice in England, as the Duchess already had a passion for these animals before Pilkington’s gift and this passion was long-standing. It is hence likely that the importation of white mice from Far East to Europe had started at least from the 1750s.

In 1768 and 1769, the British bookseller and writer Samuel Paterson (1728–1802) published (under the pseudonym Coriat Junior)^6^ an ironic account of the observations made during a two-month journey in the Netherlands in 1766 (releasing volume I in 1768^7^ and volume II in 1769). In the second volume of this work (Coriat Junior, 1769), Paterson describes the fanciness of the Dutch inns: “You cannot enter a Dutch inn, high or low, without meeting wherewith to improve, or entertain - to gratify your admiration, or risibility. A wilderness or a parterre - an aviary or a menagerie - a collection of pictures and models, or a parcel of grotesques and whim-whams.” Interestingly, among the bizarre animals that could be found in the Dutch inns (such as “a dancing marmoset” and “a squeaking Guinea-pig”), Paterson mentions a breed of white mice. Just after the comment about the exotic animals present in the Dutch inns, Paterson adds: “But I must not pass by their hoards of Japan and China Ware, some of which might enrich the cabinet of a prince.” This second note confirms that the inns in the Netherlands were receiving goods from the Far East, which could possibly have been the source of the very particular breed of white mice.

In 1787, Elizabeth Steele published a biography of Sophia Baddeley (1745–1786), a British actress and singer, who made her first stage appearance in 1764 as Ophelia in *Hamlet* and rose to the role of star at London’s Drury Lane Theatre, becoming, for her performances, beauty and extravagant lifestyle, “one of the most popular and controversial stage personalities of her time” (Highfill et al., 1973). This biography was published the year after the premature demise of Baddeley, who died, at only 41 years of age, of consumption, after having fallen into financial difficulty and having cumulated several debts (Steele, 1787). In the novel, narrated in first person, Elizabeth Steele declares to be a close friend of Sophia Baddeley and appears as a character of the story, which can hence be considered also an autobiography (Culley, 2014). In this auto/biography, Steele relays that in the 1770s one morning she met Mrs. Millidge, a dancer of the Drury Lane Theatre. While Steele was talking to her about theatrical matters, the dancer was shopping and, during the three-quarters of hour of conversation, the dancer bought, from what appears to be a pet shop, “eight white mice, with red eyes; a handsome squirrel cage to keep them in; a silver collar and bell for her cat, and new cages for all her birds” (Steele, 1787). The mice cost her 2 pounds and 12 shillings.

Travel writer Richard Twiss (1747–1821), in his third autobiographical travel book, when describing a trip to Paris that he had undertaken in summer 1792, reports that white mice were commonly sold as pets in the central area of the city, near Palais-Royal: “About the *Palais Royal* persons are frequently found who offer for sale white mice in cages; these are pretty little animals, their fur is snow white, and their eyes are red and sparkling” (Twiss, 1793).

In 1793, the British naturalist, agricultural scientist and economist James Anderson (1739–1808), at that time a key figure of the Scottish Enlightenment in Edinburgh, wrote, in an essay on within-species varieties, about albino mice and relayed having personally “seen a tame white mouse” and how once a group of recently born juvenile white mice were brought to him (Anderson, 1793a). The following year, this essay by Anderson was included, as Appendix, in the book *An Account on the Different Kinds of Sheep* by the German zoologist Peter Simon Pallas (1741–1811), who, like Anderson, adhered to the idea that the rule “to mark the distinction between a *species* and a *variety* is, that though different species of animals of the same genus may be brought to breed together, as the horse and the ass, yet the animals thus produced, are not prolific; whereas the progeny arising from an intermixture of different *varieties* of the same *species*, are themselves equally prolific as the parents from which they sprang” (Anderson, 1793b; Pallas and Anderson, 1794). Based on this principle, Anderson regarded that albino and normopigmented mice were two varieties of the same species, as he had observed that, in a brood of mice that had been brought to him, in addition to white mice there were also piebald mice, which led him to hypothesize that the parents of the brood were an albino mouse and a normopigmented mouse: “a whole nest of young mice were once brought to me, consisting of 10 or 12, which were either white or mottled, and I think few or none of them were entirely of the ordinary mouse colour. This I presume had been the progeny of a mouse probably pure white, with a mate of the usual colour” (Anderson, 1793a).

In London, white mice were sold in pet shops as early as 1798. Among the correspondence of the Monkton family from Hazelwood (Derbyshire, United Kingdom), which was collected and published in 1811 by the British historical writer and biographer Lucy Aikin (1781–1864), letter XXIII from 30 April 1798,^8^ written by Edward Monkton to his brother Robert Monkton, contains the description of a visit of Edward, at that time a little boy, to London (Monkton, 1798; Aikin, 1811). Among the attractions to be seen, Edward mentions the Tower of London,^9^ the Exeter Exchange,^10^ the Cork Models^11^ and Maillardet’s Automatic Exhibition.^12^ The boy writes that one morning, instead of going sightseeing, he was brought to see the shops of the city. After remaining enchanted by the painted glass lamps and the mirrors of a large glass shop, he then visited a pet shop, which was selling a great number of different species of birds, including parrots, parroquets, macaws, singing birds (bullfinches, canaries and Russian nightingales), Bengal sparrows, widow-birds and turtle-doves. In addition to the birds, the shop also sold a certain number of species of small mammals, including “a little French lap-dog, which was shaved to look like a lion, a brood of white mice, a hedge-hog, and a family of guinea-pigs” (Monkton, 1798; Aikin, 1811). Interestingly, the mice and guinea pigs were not only sold, but also bred by the shop. The author recounts that the guinea pigs had newborns, which were “only 2 days old, and so little that they would lie in the palm of your hand” (Monkton, 1798; Aikin, 1811).

An 1820 handbook on domestic animals, edited by the producer of instructive literature Charles Bardin (c. 1788–1841) and published in Dublin anonymously, reports that in 1819 Polito’s Menagerie^13^ was in Dublin and it exhibited white mice (Bardin, 1820). Notably, since Polito’s Menagerie was a travelling menagerie that toured across the United Kingdom and Ireland, it is likely that these albino mice were showcased in several cities of the British Isles.

In an 1831 essay on eye physiology, Jerome Van Crowninshield Smith (1800–1879), American physician and founding editor of the *Boston Medical and Surgical Journal*, in a section entitled *The Reason Why the Pupils of an Albino’s Eyes Are Red*, mentions white mice with red eyes and informs us that these animals were “brought in cages from China” (Van Crowninshield Smith, 1831). This piece of information allows us to know that in the early 1830s albino mice were still imported to the Western World from Far East. A few weeks after the publication of Van Crowninshield Smith’s essay, parts of it, including the mention of the albino mice imported from China, were popularized in the American magazine *Monthly Traveller*, where Van Crowninshield Smith was defined as “a gentleman who has already acquired an enviable reputation as a medical practitioner, a lecturer, and a man of science” (Monthly Traveller, 1831).

In 1833, the London Zoo (at that time, Gardens of the Zoological Society) enlisted in the catalogue of the exhibited animals a group of white mice. In this catalogue, the mice are described as the “well-known albino variety of the common Mouse” (Zoological Society of London, 1833). The specification “well-known” makes us understand that, by the early 1830s, in London albino mice were not a novelty any more. From the same catalogue we know that the collection of the Gardens also included one white rat, described as a “white variety of the common Norway Rat” (Zoological Society of London, 1833).

By 1841, as described by the Irish zoologist Henry Downing Richardson (1816–1849) in *The Irish Penny Journal*, albino mice had become extremely popular pets, to the point that they could be found in the vast majority of the main pet shops of London, Edinburgh and Dublin: “White mice are to be procured at most of the bird shops at Patrick’s Close, Dublin; of the wire-workers and bird-cage makers in Edinburgh; and from all the animal fanciers in London, whose residences are to be found chiefly on the New Road and about Knightsbridge. Their prices vary from one shilling to two-and-sixpence per pair, according to their age and beauty” (Richardson, 1841). In 1851, the aforementioned British journalist Henry Mayhew, in his groundbreaking survey of the habits of the Londoners, confirmed how at that time in London white mice were sold as fancy animals in the pet shop (Mayhew, 1851).

In 1864, Reverend John George Wood (1827–1889), one of the most well-known parson-naturalists and natural history popularizers of the Victorian Age, devoted an entire article to pet white mice, in which he stated that they were “great favourites with boys” and that they could “be obtained from the dealers at prices varying from sixpence to five shillings” (Wood, 1864).

In addition to albino mice, albino rats also became popular pets in 19th century United Kingdom, starting from at least the 1840s. James Rodwell’s handbook on the rat gives an account of a London rat breeder named Ostin, who around 1848^14^ started breeding white rats, “for fancy and profit,” beginning with a single couple made of a white male rat and a white female rat that he imported from Normandy, and ending up with “an immense number in cages” (Rodwell, 1858). Ostin’s son-in-law, who was trained by Ostin and subsequently opened his own business, also raised white rats, getting to have, in 1851, over 100 white rats (Rodwell, 1858). Interestingly, in addition to the albino rats, Ostin’s son-in-law experimented with the creation of other breeds of tame rats, by crossing white rats with brown and black rats, obtaining mixed strains such as the black-and-white piebald and the brown-and-white piebald (Rodwell, 1858).

By the 1850s, white rats were commonly sold as pets in London. Rodwell’s handbook states: “Instances of tame rats are by no means rare, or of their becoming gentle and attached to those who feed and caress them” (Rodwell, 1858). The handbook devotes an entire section to “Albinos, or White Rats” and reports: “In London, at the present time, these animals, being bred for fancy, are becoming very numerous, and sell at the rate of four shillings a couple” (Rodwell, 1858).

In his 1851 survey, Mayhew reports that in London there were shopkeepers selling white rats as fancy pets and for rat-baiting shows (Mayhew, 1851). Mayhew mentions in particular an animal shop that sold both fancy rats and fancy mice: “a large bird-seller, who sold also plain and fancy rats, white mice, and live hedgehogs” (Mayhew, 1851). As part of his social inquiry, Mayhew also published long interviews with two rat breeders, providing numerous details on their activity: Jimmy Shaw and Jack Black (Mayhew, 1861).

Jimmy Shaw (birth name: John James Shaw) ran, from the 1840s, the rat-baiting pit of the Blue Anchor Tavern in London. In his establishment, he kept very large colonies of rats (up to 2000 individuals) to be used in rat-baiting sport shows. Interestingly, Shaw purchased wild rats from rat-catchers, but he also bred his own colonies (Mayhew, 1861). Interviewed by Mayhew, he declared: “At time I bred rats very largely.” At the end of the interview, Shaw showed Mayhew “some very curious specimens of tame rats—some piebald, and others quite white, with pink eyes, which he kept in cages,” and explained that he was performing artificial selection trials to produce white rats (Mayhew, 1861).

Jack Black, on the other hand, was a rat-catcher, that eventually became an animal seller, as described by Black himself in the interview that he granted to Mayhew (Mayhew, 1861). At the beginning of the interview, Black declared that he had been rat-catching for almost 35 years. He then recounted the history of his professional career, which is faithfully reported verbatim by Mayhew in the third volume of his social survey. Black started catching rats in Regent’s Park in London when he was a little child, at the age of 9. At about 10 years of age, Black began using ferrets to hunt them and selling the wild-caught rats to people who carried on rat-baiting sports. He continued this rat-catching activity up to the age of 13 or 14. In these years, he also bred ferrets and sold them in great numbers. At about 15 years of age, Black turned to bird-fancying, raising and selling linnets. In the interview to Mayhew, he declared: “I was very fond of the sombre linnet. I was very successful in raising them, and sold them for a deal of money.” Subsequently, he began breeding and training monkeys: “I’ve ris and trained monkeys by shoals. Some of mine is about now in shows exhibiting.” He also resumed his rat-catching activity and in the 1840s (15 years before the interview)^15^ he started working as rat destroyer for the Government. One year later, he started going “about the streets exhibiting with rats.” He roamed London with a picturesque costume and a cart with rats painted on it, offering his pest control services to anybody who needed them. He “used to wear a costume of white leather breeches, and a green coat and scarlet waist-kit,” bearing a hat with a golden band around it and a belt with four white rats painted on it. In his street exhibitions, Black carried with him some rats in cages and showcased his ability in handling the rats: “I used to take out the rats, and put them outside the cage; and used to begin the show by putting rats inside my shirt next my buzzum, or in my coat and breeches pockets, or on my shoulder – in fact, all about me, anywhere. The people would stand to see me take up rats without being bit.” Black had prepared a poisonous composition for rat extermination and was selling it from the cart. In order to prove the effectiveness of his composition, he administered it to a rat and showed its lethality “afore the people’s own eyes.” Due to his pest control activity for the Government, Black became popular as “the Queen’s ratcatcher.” When interviewed by Mayhew, Black was still keeping the bills of his assignments, which he showed to Mayhew, who could ascertain that they reported the formula: “V. R. Rat and mole destroyer to Her Majesty,”^16^ However, Black did not only exterminate rats. He also kept many, which he sold for 3 pence each. Black declared to Mayhew: “I’ve got cages of iron-wire, which I made myself, which will hold 1,600 rats at a time, and I’ve had these cages piled up with rats, solid like.” Importantly, Black did not simply sell wild-caught rats for rat-baiting sports, but, at least from the 1850s, he also bred rats and started selecting specific varieties such as piebald rats, which he sold as domestic pets: “I’ve bred the finest collection of pied rats which has ever been knowed in the world. I had above eleven hundred of them – all variegated rats […] and all of them in the first instance bred from the Norwegian and the white rat.” Black caught his first albino rat in Hampstead (a district in northern London) and, through a series of crossings, he obtained six varieties of piebald rats: “fawn and white, black and white, brown and white, red and white, blue-black and white, black-white and red.” The new rat strains created by Black became a sensation: “People come from all parts of London to see them rats, and I supplied near all the “happy families” with them.” These piebald rats “got very tame” and made very good pets: “I’ve sold many to ladies for keeping in squirrel cages.” Black sold many of these fancy rats directly to private citizens, but he also supplied pet shops, such as the one in Leicester Square. At the time of the interview, Black lived in Battersea (a district in southwestern London) and continued to sell animals, including birds and fish for vivaria. Additionally, he had moved his “attention to everything connected to animals,” for instance preparing veterinary remedies to cure the mange in horses and dogs.

Zoologist Francis Trevelyan Buckland (1826–1880), in his *Curiosities of Natural History*, recollects how in the 1850s tame albino rats were brought up to the North Pole. Indeed, in 1853, when Captain Edward Augustus (1820–1894) left for the North Pole with the ship Phoenix, Buckland’s sister “gave him three white pet rats which were quite tame. These three rats went to the North Pole in a cage specially provided for them, and became quite favourites on board. One of them died of an abscess in the back, the second was given away to one of the officers belonging to a ship that was at Melville Island, and the third came back to England sound and well” (Buckland, 1857).

By the 1870s, the habit of keeping albino rats became further consolidated in Europe. Indeed, an 1872 issue of the magazine *Every Sunday* informs us that albino rats were particularly popular among the ladies of Paris: “Parisian fashionable ladies are getting tired of scented pugs, possibly because the duty on them has been doubled, and now take a kind of small white rat for a pet” (Every Saturday, 1872). In 1875, the British magazine Cassell’s Family Magazine defined the white rat as “the dearest of pets” (Cassell's Family Magazine, 1875). In 1880, the British physician and novelist Gordon Stables (1840–1910) wrote an article for the children magazine *The Boy’s Own Paper* entitled *Rats and Mice as Pets*, in which he enthusiastically praised the neatness of albino rats (“There is no more cleanly animal in the world than the white rat”) and provided detailed suggestions for their care (Stables, 1880). Notably, in the title of Stables’s article “Rats” precedes “Mice,” which indicates how, by 1880, rats were no longer second to mice as pet rodents. Just 4 years later, in 1884, the British journalist Thomas Heath Joyce (1850–1925), in an article on pets, described the white rat as “one of the tamest and most intelligent pets possible” (Joyce, 1884).

### 4.3 Showcasing interspecies social bonding in the 19th century: “happy family” shows

In the 19th century, the exhibitions of multiple different species, including predators and preys, living together amicably in the same environment became a popular phenomenon, known as “Happy Family” shows. Such shows began in the United Kingdom and in Ireland in the 1810s and were imported in the United States in the 1840s.

Bardin’s handbook on domestic animals relays that, although the cat has a natural hostility (“animosity”) towards mice and birds, training (“education”) “can conquer all its natural propensities” (Bardin, 1820). As an example of this, Bardin reports that, when Polito’s travelling menagerie was in Dublin 1819, a happy family was exhibited: “In a large cage, there are living together in the most amicable manner, 2 guinea pigs, several birds of the linnet and canary species; and what is still more extraordinary, two white mice” (Bardin, 1820). The process to achieve this result is then described: “The keeper says, the cat was first put amongst this collection when a very young kitten – so that it most probably became familiarized to its companions, before it had the power of shewing its malignity. – The conquest over its natural disposition, however, appears to be complete, since it refuses to injure brown mice, which are frequently presented to it by way of experiment” (Bardin, 1820).

Happy Families are also mentioned in an 1848 novel by the Irish writer Anna Maria Hall (1800–1881), who often published under the pseudonym “Mrs. S. C. Hall,” in reference to one shown in Dublin (Hall, 1848). In the novel, Hall describes the happy family as “That cage full of birds and animals; cats, mice, hawks, and finches, all living lovingly together: they call that the happy family” (Hall, 1848). In the same years, happy family shows were popular also in London. Indeed, under the section devoted to “The Street Showmen,” Mayhew reports the Happy Family shows as instances of exhibition of extraordinary animals (Mayhew, 1851).

British animal trainer and showman John Austin (1790/1–1852), also known as Ostin, was the first to create the Happy Family shows. Austin started Happy Family exhibits featuring a cat and white mice in Manchester in the early 1810s and in London in 1815, adding to his achievements Happy Family shows with white rats in London from the late 1840s (The Economist and General Adviser, 1824; Lemon and Mayhew, 1841; Rodwell, 1858; Mayhew, 1861).

Rodwell’s handbook on the rat describes how the rat breeder and showman “Ostin,” started breeding white rats from 1848 and employed them to set up a Happy Family show, which in 1851 could be visited daily at the foot of Waterloo Bridge. Moreover, Ostin initiated in the art of animal breeding and training also his son-in-law, who opened his own business and exhibited his happy family every evening in Regent Street, having, in 1851, over 100 white rats.

In 1851, Rodwell visited personally, and watched amused for hours, the Happy Family of Regent Street, which he describes in detail in his 1858 handbook (Rodwell, 1858). According to Rodwell, in a large cage of 6 ft. by 4 ft. (1.8 m by 1.2 m) with a height of 4.5 ft. (1.4 m), a most singular group of animals was living together in harmony: jackdaws, magpies, hawks, owls, starlings, pigeons, a white cat, 5 white kittens, 36 white rats, many purely brown and purely black rats, many piebald rats, guinea-pigs, rabbits, a white ferret, a white-and-black dog and a monkey (Rodwell, 1858). Interestingly, the monkey (a male named Jacko) developed an extraordinary attachment to one of the young white rats. Rodwell relates that, apparently, Jacko “adopted it as his own, as he nurses it and fondles over it just as a mother would over her child, and the rat is perfectly conscious of the attachment, and is quite attached to the monkey.” Rodwell witnessed himself an example of this amazing attachment. Indeed, when the monkey received a biscuit, he “immediately caught his favourite, and, placing it in his lap, gave it a piece, and then had a mouthful himself.”

Henry Mayhew provides more information about the rat breeder mentioned by Rodwell as “Ostin,” referring to him with his birth name John Austin (Mayhew, 1861). From Mayhew we learn that John Austin was originally a stocking-weaver in Nottingham. He started breeding white mice and creating “Happy Families” in which the mice lived in harmony together with a cat. When he succeeded, he took the animals with him and exhibited them in Manchester. Subsequently, Austin moved from Nottingham to London and began exhibiting the animals there. He started his Happy Family show in Waterloo Road, at a time when the Waterloo Bridge had not been built yet. The show was a tremendous success: “Everybody who passed gave him money. Noblemen and gentlepeople came far and near to see the sight.” According to what is reported by Mayhew, John Austin began exhibiting his Happy Family show in London in 1815 and continued “for 36 years all but 5 months” until he closed his activity, short before his death, which occurred in February 1852. In 1833, Austin had the honor to exhibit before Queen Victoria (1819–1901), at that time Princess: “She sent for him expressly, and he went to Buckingham Palace, he would never tell anybody what she gave him; but everybody considered that he had been handsomely rewarded.” After having presented his show at Buckingham Palace, Austin was invited to exhibit it at the Mechanics’ Institution in Hull and at the Mechanics’ Institution in Liverpool, and travelled across England with his show for many other institutions.

In the early 1850s, when Austin was old, many were imitating him and there were at least five Happy Family shows in London (Mayhew, 1861). Mayhew additionally describes the activity of a second animal trainer, a man that had been assistant to John Austin for 8 years and subsequently opened his own Happy Family show in London, likely the “son-in-law” mentioned by Rodwell (Mayhew, 1861). Austin’s assistant launched his show with a collection of animals comprising two monkeys, white rats, piebald rats, cats, dogs, hawks, magpies, ferrets and a coatimundi from South America. In 1851 his stand was in Regent Street, by the corner with Castle Street. Subsequently, he shifted to Tower Hill, where he remained for more than a year and a half, and then he moved from place to place for a while, until he returned to the original spot of John Austin, Waterloo Road, where he stayed for a few months. At the time that Mayhew interviewed him (1856),^17^ the man was still maintaining a Happy Family and showcasing it.

**Figure 4:**
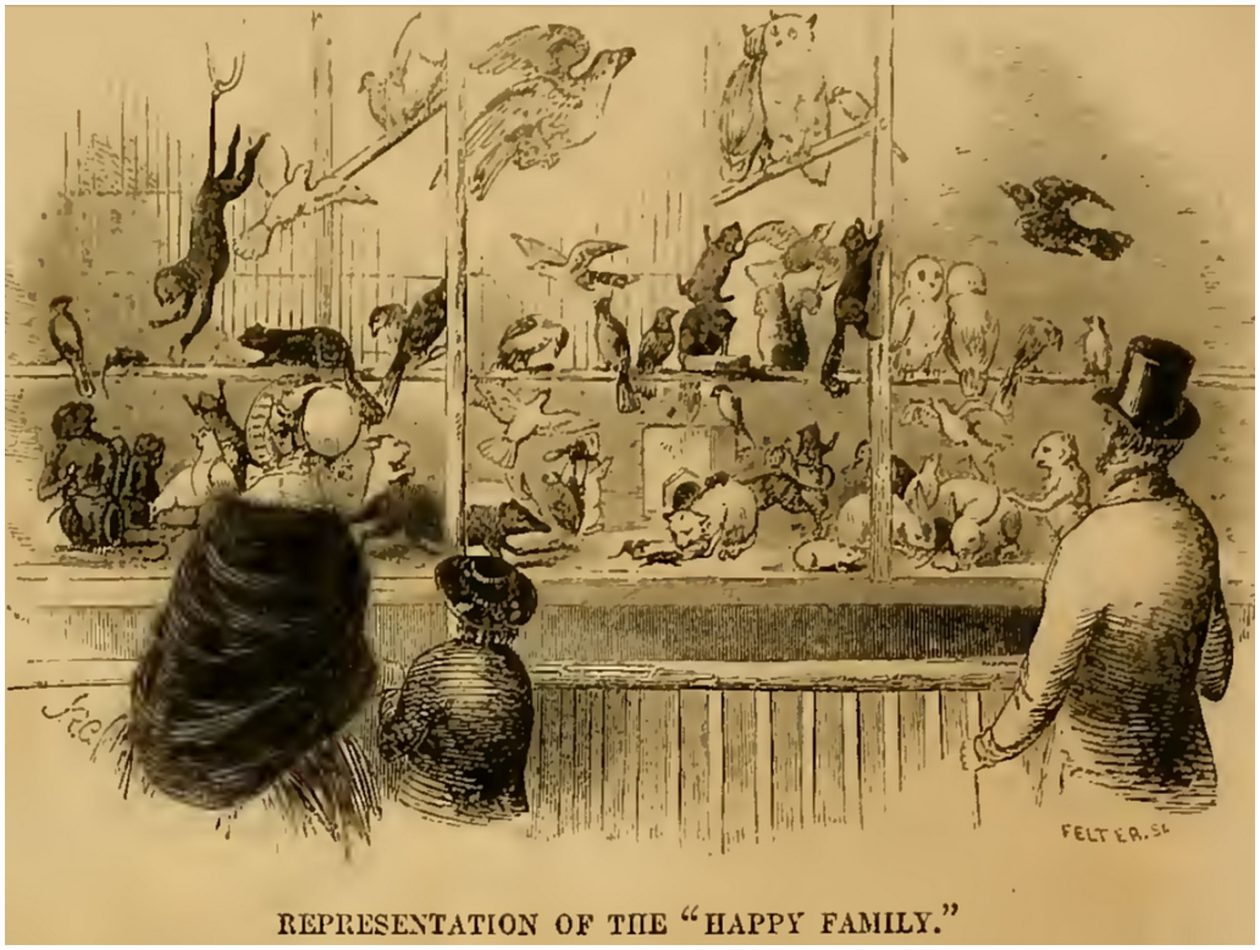
Barnum’s Happy Family exhibit. The figure depicts the Happy Family exhibit of Barnum’s American Museum in New York, as represented in the Boston illustrated magazine *Gleason’s Pictorial* in January 1853 (Gleason’s Pictorial, 1853) by the American artist John Daniel Felter (1825–1914).

Happy Family exhibits were first brought to the United States by the famous showman Phineas Taylor Barnum (1810–1891), better known as P. T. Barnum. In his autobiography, Barnum describes a visit to Coventry (Warwickshire, United Kingdom) in late 1844 and recounts: “we visited an exhibition called the “Happy Family,” consisting of about two hundred birds and animals of opposite natures and propensities, all living in harmony together in one cage. This exhibition was so remarkable that I bought it for $2,500, (£500,) and hired the proprietor to accompany it to New-York, where it has ever since been an attractive feature in my Museum” (Barnum, 1855). In 1853, *Gleason’s Pictorial*, the first illustrated magazine in America, devoted an article to Barnum’s American Museum in New York (Gleason’s Pictorial, 1853), providing a graphical depiction of the Happy Family, which we reproduce in Figure 4. In 1869, the guide book to Barnum’s American Museum still enlisted the Happy Family in its catalogue (Barnum's American Museum, 1869). Interestingly, in Barnum’s Happy Family many American species were added and in 1869 the exhibit comprised “8 doves, 4 owls, 10 rats, 2 cats, 2 dogs, 1 hawk, 3 rabbits, 1 rooster, 8 Guinea Pigs, 1 Raccoon, 2 Cavas, 1 Cuba Rat, 3 Ant Eaters, 7 Monkeys, 2 Woodchucks, 1 Opossum, 1 Armadilla, &c., &c” (Barnum's American Museum, 1869).

### 4.4 Albino rodents in early scientific research

Rodents, especially rats and mice, are currently the most commonly employed animal models in biomedical science (Zhao et al., 2007; Hickman et al., 2017; Voikar, 2020). In particular, albino rats and albino mice have become icons of the laboratory animal model, with the albino rat considered as the prototype of the laboratory rat (Logan, 1999, 2005; Kuramoto, 2012) and albino mice among the five most widely used mouse models (Burlikowska et al., 2020). Nevertheless, the history of the origin of albino rats and mice in scientific research is little known. In this section, a history of these albino rodents in early scientific research is traced.

Rodents have been used as test subjects for biomedical experimentation since the 1650s (d'Isa et al., 2024a). However, the first rodents employed for experimentation were wild-caught normopigmented individuals. On the other hand, albino rodents started to be used only later, from the early 19th century.

The very first use of albino rodents in scientific research dates to more than 200 years ago and, interestingly, it was not motivated by the color of their fur but of their eyes. Indeed, in the 1810s, the French physiologist François Magendie (1783–1855), pioneer of experimental medicine, used albino mice as animal model in studies on the physiology of vision, exploiting the absence of pigmentation in the eye and the consequent transparency of the cornea to observe more easily the images projected by luminous objects on the retina (Magendie, 1816; English translation: Magendie, 1825).

Few years later, in 1821, the Swiss pharmacist and naturalist Jean-Antoine Colladon (1755–1830), founding member of the Société de Physique et d’Histoire Naturelle de Geneve (from 1790) and founding member of the Société Helvétique des Sciences Naturelles (from 1815), carried out experiments on hybridization, crossbreeding white mice with grey mice and finding, surprisingly, that no mice with mixed coats were generated (Edwards, 1829; de Morsier and Cramer, 1959; Tecoz, 1959; de Morsier, 1966; Corcos, 1968). In 1829, French physiologist and ethnologist William Frédéric Edwards (1777–1842), a former pupil and laboratory assistant of Magendie, related that Colladon, “to increase the experiences on breed crossings and extend our ideas on this subject, raised a large number of white mice and grey mice. He carefully studied their habits and found a way to make them reproduce by crossings. He then began a long series of experiments by always mating a grey mouse with a white mouse. What result do you expect? That there were often mix-ups. No, never. Each individual of the new litter was either entirely grey or entirely white, with the other characteristics of the pure race; no mixed race, no variegation, nothing intermediate” (Edwards, 1829).^18^ From the proceedings of the Société de Physique et d’Histoire Naturelle de Geneve, we learn that Colladon first presented his results in a lecture held at the society on 3 May 1821 (de Morsier and Cramer, 1959). In 1822 Colladon presented, at the same society, a follow-up investigating whether the lighting condition (light or darkness) of the housed mice could affect the color of their offspring, since it was believed that albinos are more likely generated if the parents are living in the dark (Girtanner, 1796; Thornton, 1800; Trent, 1800). Colladon reported that the white color remained absolutely stable across generations, regardless of the lighting condition (de Morsier and Cramer, 1959).

Colladon also provided albino mice to the Swiss physician Jean-Louis Prévost (1790–1850) and to the French chemist Jean André Dumas (1800–1884), supervisor of the laboratory of the pharmacy Le Royer of Geneve, for their comparative microscopic study of spermatic cells across different animal species. Interestingly, Prévost and Dumas found that the spermatozoa (“animalcules spermatiques”) of white mice were remarkable, as, compared with the ones of other mammals (guinea pig, hedgehog, sheep, horse, cat and dog) and of non-mammals (sparrow, cock, duck, viper, salamander and snail), they were the only ones with multiple bright spots on the head of the spermatic cell (Prévost and Dumas, 1821). The same authors subsequently carried out a direct comparison between white mice and grey mice (which had been obtained from Colladon) and discovered that their spermatozoa are identical, as for the length, the shape and the spots (Prévost and Dumas, 1824). Prévost and Dumas also observed that the peculiar spotted head of the spermatozoon was a characteristic shared by white mouse, grey mouse and rat (Prévost and Dumas, 1824). Additionally, in their second account, Prévost and Dumas report having witnessed the experiments of Colladon and confirm that, according to his findings, the crossing of white and grey mice does not generate mice with mixed fur, specifying that this is true also if the crossings are made between members of the first generation, or the second, or the third (Prévost and Dumas, 1824). The authors also provide information regarding the timing of Colladon’s experiments. They relay that while they were working on their research on the anatomy of the spermatozoa of white mice, Colladon communicated at the Society the results of his experiments on the crossbreeding of white mice, which he had been performing from “some years” earlier (Prévost and Dumas, 1824). Since Colladon communicated his results at the Society in 1821 and 1822, this means that Colladon initiated the colony of white mice at least in the late 1810. Importantly, Colladon’s colony of artificially selected albino mice, started in Geneve in the 1810s, is the first mouse line ever developed for exclusively scientific purposes.

**Figure 5:**
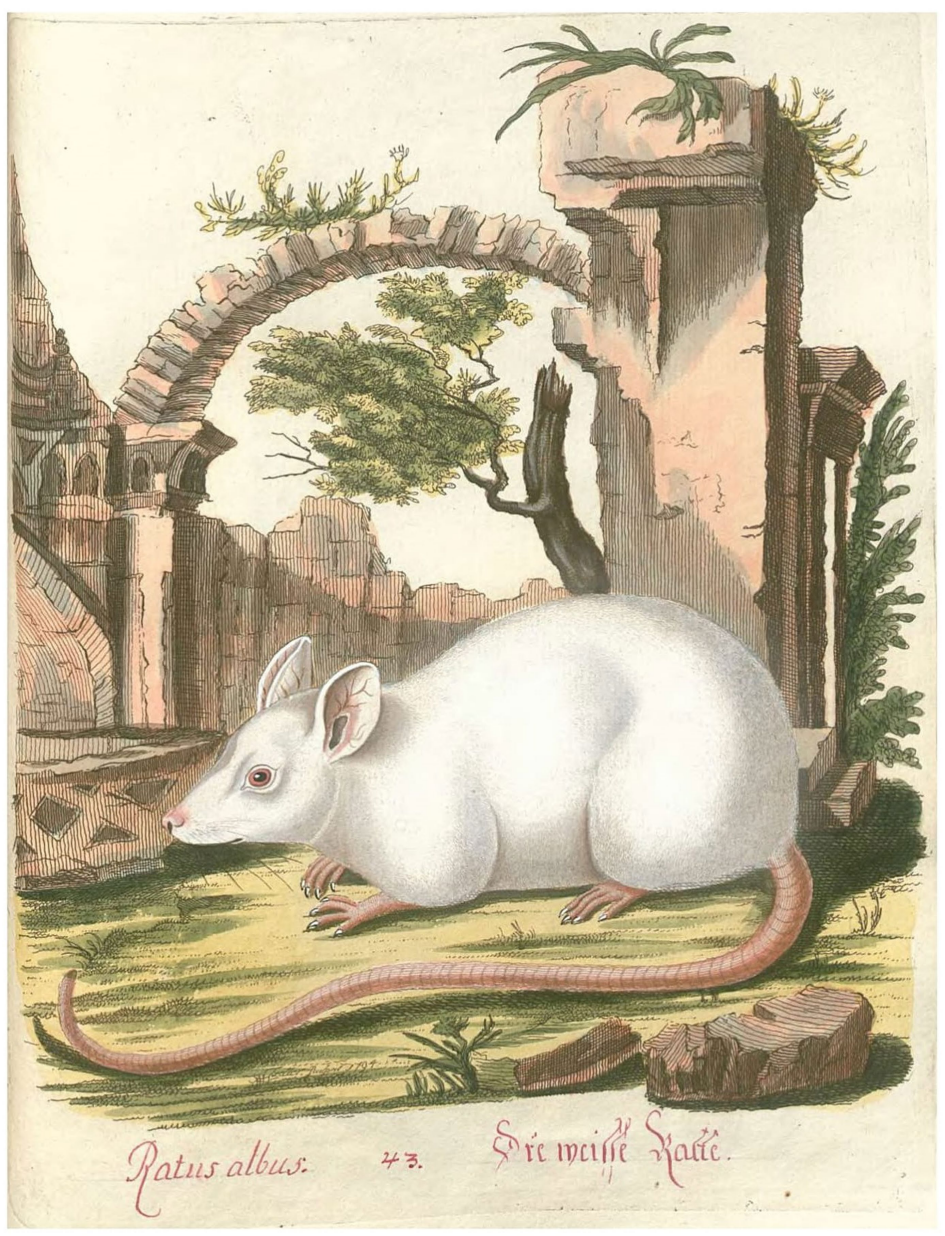
The first graphical representation of the albino rat in Western scientific literature. In 1794 Spalowsky provides a depiction of the albino rat in his *Naturgeschichte der vierfüßigen Thiere* (Natural History of Quadruped Animals). Spalowsky defines this animal “Ratus albus” in Latin and “weisse Ratte” in German, both phrases meaning “white rat.” The illustration is Plate 43 of the first volume of Spalowsky’s zoological encyclopedia (Spalowsky, 1794).

The first scientific research study on a captive albino rat was performed in the 1790s by the Polish-Czech naturalist Joachim Johann Nepomuk Spalowsky (1752–1797), at that time state physician in Vienna. Notably, Spalowsky is also the first to provide a graphical depiction of the albino rat in Western scientific literature. In 1794, Spalowsky reported having personally found a white rat, of which he presented a detailed illustration in color (Spalowsky, 1794). We reproduce this illustration in Figure 5. At that time, the origin of white rats was unknown and some naturalists were claiming that the observed cases could have been just old rats, which had developed a white fur as a result of senescence. However, Spalowsky refuted this hypothesis, relating that he had collected this rat and another four from their nest when they were little juveniles still completely blind.^19^ Additionally, as a further proof, he argued that the eyes of the white rats were red, which could not come from an aging process. Spalowsky specified that these white rats could be found in the fissures of old walls of abandoned dilapidated buildings, especially after a very dry summer. Since Spalowsky collected the white rat from its nest when it was a juvenile and raised it in captivity until it could be represented in the illustration as an adult, Spalowsky’s work is the first scientific research study of a captive albino rat ever published.

The second scientific study of a captive albino rat is the study by Samuel Moss, which was carried out from 1822 to 1825, and published in 1836 (Moss, 1836). Importantly, the work by Moss is the first behavioral study on a captive rat ever published. However, the observational studies by Spalowsky and by Moss were, respectively, zoological and ethological/psychological, without employment of the white rat as an animal model for experimentation.

Albino rats started to be used as animal models in experimental medicine at least from the 1850s, in both the United Kingdom and France. British chemist and toxicologist John Horsley (1819–1889), from the County Analyst’s Laboratory^20^ of the Police-Station of Cheltenham, was the first to use white rats as animal model for experimentation. Horsley experimented on the effects of strychnine on white rats, submitting his results in an article dated 24 July 1856, which was first read, in August 1856, at the 26th Meeting of the British Association for the Advancement of Science held in Cheltenham, and was subsequently published in a medical journal on 6 September 1856 (Horsley, 1856).

In France, the first laboratory to employ albino rats for scientific experimentation is the laboratory of comparative physiology of Pierre Flourens (1794–1867) at the Muséum d’Histoire Naturelle in Paris. In 1856, in Flourens’s laboratory, the British physician Augustus Volner Waller (1816–1870), following Magendie’s example, employed albino rats to perform microscopic observations on the circulation of blood in the vessels of the eye, seen through transparency in the live animal (Waller, 1856). In the same year, another member of the laboratory, the French physician Edmé Félix Alfred Vulpian (1826–1887), who had previously discovered adrenaline, extracted adrenaline from albino rats and observed that it turned red when exposed to air, discovering hence adrenochrome, which is the biomolecule resulting from the oxidation of adrenaline (Vulpian, 1856). Also in 1856, the French physician and pioneer of experimental regenerative medicine Jean-Marie Philipeaux (1809–1892), assistant to the holder of the Chair of Comparative Physiology Pierre Flourens, used albino rats to evaluate the effect of the removal of the supra-renal capsules (Philipeaux, 1856). These three papers from the Flourens laboratory were first presented at the Acadèmie des Sciences in Paris, under the presidency of the zoologist Isidore Geoffroy Saint-Hilaire (1805–1861). Waller and Vulpian presented their papers in the session of 29 September 1856, while Philipeaux presented his in the session of 10 November 1856.

**Figure 6:**
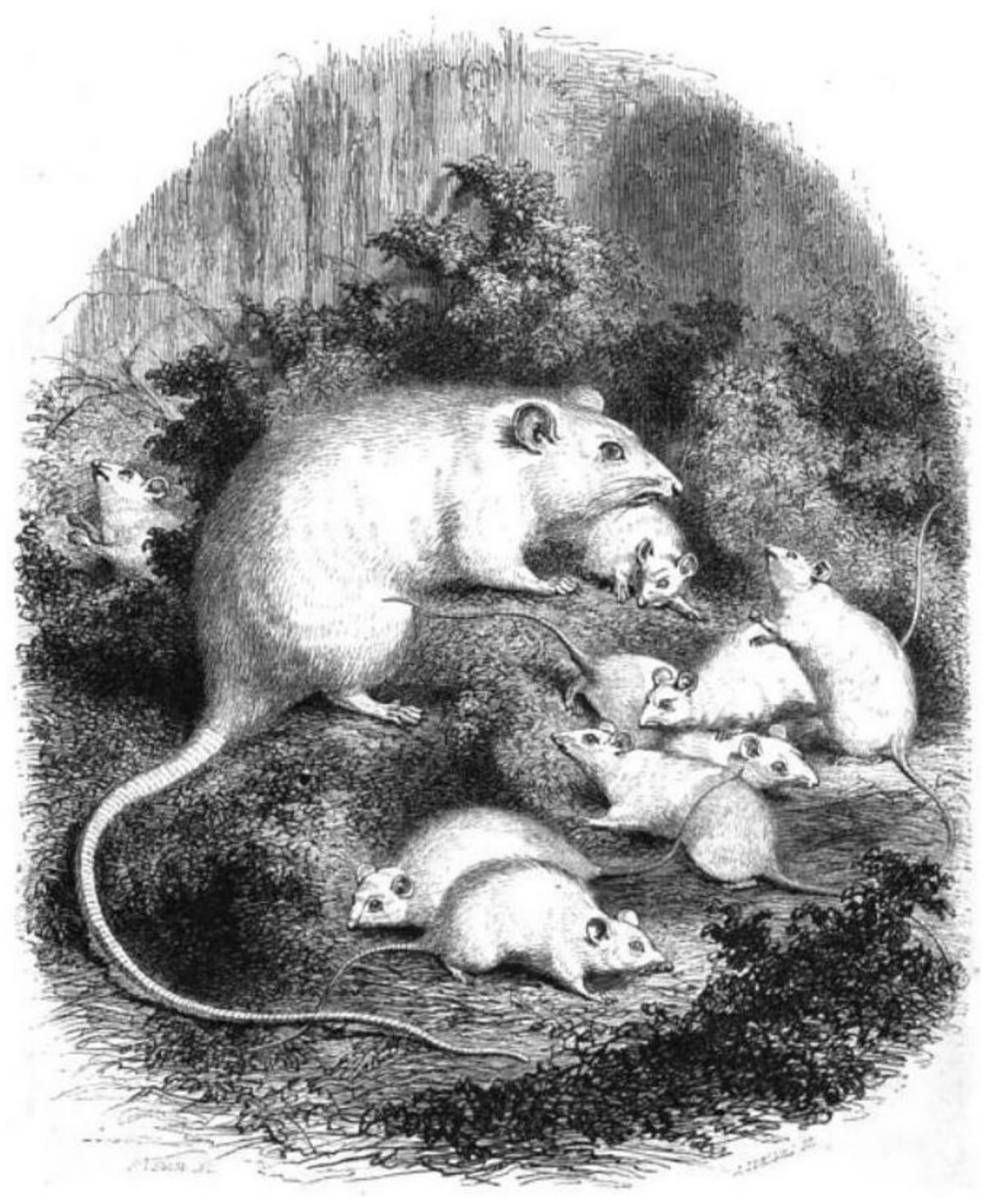
Albino rat colony bred at the Jardin des Plantes of the Muséum d’Histoire Naturelle of Paris. The image portrays a female albino rat with its offspring in the rat breeding colony of the Jardin des Plantes of Paris as represented in Le Magasin Pittoresque, the first illustrated magazine in France (Le Magasin Pittoresque, 1856). Image drawn from life in 1856 by the British artist William Henry Freeman (born around 1806 and active until 1875). Note: although in the caption of the original figure the animals are described as “souris” (mice), this is a mistake of the author of the article, as the species can be clearly identified as rat from the graphical representation of the tail.

Interestingly, in the year of the publication of these three works from the Flourens laboratory (1856), the menagerie of the Jardin des Plantes (which from 1793 was under the Muséum d’Histoire Naturelle and basically represented the living section of the collection of the museum) was breeding colonies of white mice, white rats and black-and-white rats obtained from the crossing of black rats with white rats (Le Magasin Pittoresque, 1856; Bazin, 2001). An 1856 drawing from life depicts the colony of albino rats of the Jardin des Plantes of the Muséum d’Histoire Naturelle of Paris (Figure 6). It is extremely likely that Flourens’s laboratory, which was part of the Muséum d’Histoire Naturelle, obtained the albino rats of its study from the colony of the Jardin des Plantes (Bazin, 2001). This colony of white rats, the first of a scientific institution, was established in late 1853, as described by the zoologist Auguste Henri André Duméril (1812–1870), herpetologist at the Muséum d’Histoire Naturelle, in an account, which the author dates “January 1854,” on the observations collected from the reptile menagerie of the Jardin des Plantes (Duméril, 1854). In this account, when outlining the diet of the snakes, Duméril recounts that initially they fed these reptiles with wild-caught rats, but recently they had started breeding white rats: “The white rat, which reproduces and raises very easily in captivity, will undoubtedly provide, in a few months, a valuable resource. Just from 4 weeks ago, we obtained a very numerous offspring from broods which we raise with care for this purpose, to give rats to the snakes that, in this short period, have eagerly taken 12, as well as 15 piebald rats that recently we have also been raising in cages where their reproduction seems to be abundant like that of the white rats” (Duméril, 1854).^21^

British physician George Harley (1829–1896), lecturer of practical physiology at University College London (UCL), as a follow up to the studies of the Flourens laboratory, performed further experimental inquiries into the function of the supra-renal capsules in London and in 1857 he employed as animal models, in addition to white mice, two different strains of rats: albino rats and white-and-brown piebald rats (Harley, 1857, 1858).

In the early 1860s, the employment of albino rats in experimental research was further consolidated. Shortly after Harley, in 1860, the British physician William Scovell Savory (1826–1895), lecturer on general anatomy and physiology at St. Bartolomew’s Hospital in London, performed experiments using albino rats and two mixed strains (the white-and-brown and the white-and-black) to test the effects of a low-nitrogen diet (Savory, 1863). In 1861, Savory carried out a new series of diet experiments on the same three rat strains measuring the levels of nitrogen in the urine of rats under low-nitrogen diet or low-fat meat diet (Savory, 1863).

In 1862, Flourens in person performed an ingenious experiment using white rats to demonstrate that the diet of the mother can affect the composition of her milk and hence affect the organism of the suckling newborns (Flourens, 1862). Flourens added in the food of a female rat, a nursing mother, an extract of common madder (*Rubia tinctorum*), which is used to obtain a red dye. During their first days of life, before weaning, rats are unable to eat solid food and they only suck milk from the mother. Flourens found that, although the mother’s milk appeared white as usual, the osseous tissues of the newborns became red after the newborns had been nursed by the female rat fed with the madder-enriched diet.

In July 1863, the French microbiologist Casimir-Joseph Davaine (1812–1882), who had previously demonstrated the transmissibility of anthrax disease in the sheep, evaluated the effect of anthrax injection in the white rat (Davaine, 1863). Interestingly, Davaine found a species-specific resistance to the toxin. In particular, anthrax-injected rabbits died between 17 and 63 h from the inoculation. On the other side, the white rat, even after two injections of anthrax, was still alive after 5 days and its blood did not show abnormalities.

In 1864, the French physiologist Claude Bernard (1813–1878) tested the effect of the injection of three opium alkaloids (codeine, morphine and narceine) on the behavior of white rats (Bernard, 1864, 1865). In particular, Bernard evaluated the narcotic effect of these substances, finding that, if three albino rats received each one of the three substances and were then placed in the same cage, they all feel asleep. However, if the bars of the cage very made vibrate slightly, only the rat treated with codeine woke up. On the other hand, if the bars were made vibrate more strongly, both the codeine rat and the morphine rat startled (but the first much more than the second), while the narceine rat did not move and continued to sleep.

In the German Empire, between 1877 and 1885, the biologist Hugo Crampe from the Agricultural School of Proskau (now Prószków in Poland) published a series of breeding experiments based on the crossing of albino and grey rats (Crampe, 1877, 1883, 1884, 1885). In these artificial selection experiments, Crampe practiced close inbreeding of rats up to 17 consecutive generations. Notably, Crampe’s line is the first line of inbred rats ever created for an exclusively scientific purpose. Indeed, the rat line of the Muséum d’Histoire Naturelle of Paris was used also for scientific experimentation, but it was originally established to provide food for the snakes of the reptilarium of the Jardin des Plantes. Conversely, Crampe’s line was from the start initiated for scientific research purposes.

In the United States, the first to use albino rats for laboratory science was the Swiss psychiatrist and neurophysiologist Adolf Meyer (1866–1850), who employed them in 1893 within a practical course of neuroanatomy at the University of Chicago (Logan, 1999, 2005). Colin Campbell Stewart (1873–1944), from Clark University in Worchester, was the first in the United States to use albino rats for experimentation, starting from 1895 (Miles, 1930) and publishing, 3 years later, the first American study featuring laboratory albino rats, a study which investigated the effects of alcohol, barometric pressure and type of diet on voluntary motor activity (Stewart, 1898).

In 1897, at the meeting of the American Neurological Association, Meyer suggested to the American neurologist and physiologist Henry Herbert Donaldson (1857–1938) the albino rat as the perfect test model for experimental neurology research (Logan, 1999, 2005). Donaldson, who at that time was at the University of Chicago, recognized the value of Meyer’s suggestion and from then on he employed albino rats in most of the experimental projects of his laboratory (Conklin, 1939; Logan, 1999, 2005).

In 1906 Donaldson moved from the University of Chicago to the Wistar Institute of Anatomy and Biology in Philadelphia, bringing with him four pairs of albino rats (Logan, 1999). At the Wistar Institute, for which he had been named scientific director, Donaldson, together with his pupil, the Japanese biologist Shinkishi Hatai (1876–1963), established a colony of white rats and produced a first line of moderately inbred albino rats (Donaldson, 1915; Tocher Clause, 1993; Logan, 1999). Subsequently, in 1909, the American geneticist Helen Dean King (1869–1955), who had joined the Wistar Institute in February of that year, in collaboration with Donaldson, created, through close inbreeding, a strain of artificially selected genetically homogeneous albino rats which would eventually become known as the King Albino rat strain (Tocher Clause, 1993; Suckow and Baker, 2020). Importantly, these albino rats were there first standardized laboratory animal ever created. In the same year, Donaldson definitively affirmed the perfect suitability of the albino rat for biomedical research: “As we have progressed with our studies on this animal, it has become increasingly evident that the choice was a fortunate one, as the albino rat is easy to keep, breeds freely, bears young that are both numerous and immature, and is also responsive to changes in its environment as well as being easily trained. It would be hard to find another animal that combined so many virtues in so compact and pleasing a form” (Donaldson, 1909). From 1907, the Wistar Institute made Wistar rats commercially available and started to supply them to other scientific institutions, which allowed the albino rat to become a common standard for animal testing in laboratories worldwide (Tocher Clause, 1993). At present, more than half of all the rat strains employed in scientific research, including the popular Sprague Dawley rats and the Long-Evans rats, derive from the Wistar rats (Brower et al., 2015; Hedrich, 2020).

### 4.5 Abundance of sightings of albino rats in the 19th century

Finding albino varieties of rodents freely living in urban environments was not so uncommon in the 19th century in the British Isles. For instance, Moss states that after the first white rat was found in 1822 in the farm containing the kennel and the stables of Colonel Berkeley, other white rats were observed in Cheltenham. Indeed, in the summer of 1824 (Griffith, 1826), the kennel and the stables of the Colonel were demolished to leave space for the pleasure grounds of Pittville Park, which opened in 1830 as a private urban park for Pittville (Rowbotham and Waller, 2004), the new residential neighborhood created by the lawyer and real estate developer Joseph Pitt (1759–1842). According to Moss, after the demolition of Colonel Berkeley’s kennel and stables, the “white rats were dispersed, and took up their quarters in various parts of the town” (Moss, 1836). In the town, the white rats survived for more than a year. At the end of 1825 two or three piebald rats were found and after that no entirely nor partly white rat was spotted, suggesting that the Cheltenham colony of white rats had become extinct.

Moss also reports that a considerable number of white rats could be found in Nelson Street in Bristol (Moss, 1836). He states that the white rats of Bristol had been “brought there, probably, in some of the trading vessels.” Additionally, Moss expresses the belief that the Cheltenham colony of white rats could share the same origin, deriving from the Bristol colony: “from thence [Bristol] it is likely that they found their way to Cheltenham.”

In 1823, the British ornithologist John Scales (1794–1884), received a letter from his friend Robert Hamond who was in Cheltenham, in which Hamond recounted that a few days earlier five white rats were caught “at a farm-house near Cheltenham” and that he obtained one, which he would bring to Norfolk to show to Scales (Hamon, 1823; Newton, 1885). In December 1825, a white rat was caught in the propriety of Mr. Luccock in Diss, Norfolk (Chambers, 1829).

In 1830, at the Cambridge Philosophical Society of the University of Cambridge, Reverend Henry Fardell (1795–1854) presented a white rat and gifted it to the Society (The London Literary Gazette, 1830). The white rat was exhibited in the meeting of 13 December 1830, with the Cambridge University professor and Dean of Peterborough Thomas Turton (1780–1864) as chairman.

In 1837, in a kennel of Belmont (County Wexford, Ireland), white rats were often seen (Thomson, 1856). The eyes of these rats were pink and the shape of their body was different from that of the common rat, being longer and narrower (Thomson, 1856). In 1846, white rats were found in Wymeswold in England (MacLean, 1846).

In 1847, the Irish zoologist Henry Downing Richardson (1816–1849) relayed that, some years earlier, in Greenock in Scotland a great number of white rats could be found, especially about the shipping areas (Richardson, 1847). Moreover, Richardson recollected that many specimens of white rats had been sent to him and that he kept one white rat “as a pet for a considerable time” (Richardson, 1847).

In *Historia Naturalis Orcadensis*, a zoological catalogue of the animals living in the Orkney Islands, published by the naturalist and explorer William Balfour Baikie (1825–1864) together with Robert Heddle (1826–1860) in 1848, the authors, when mentioning the common house mouse, report that: “Examples of the Albino variety have been frequently caught in the neighborhood of Kirkwall, originating, no doubt, from the escape of tame white mice” (Baikie and Heddle, 1848).

In 1851, a colony of white rats was discovered in the Ainsworth Colliery near Bury in England (Morning Chronicle, 1851; Rodwell, 1858). In his 1858 handbook on the rat, Rodwell, after reporting having seen the colony of a London breeder of white rats, which he describes as pink-eyed and smaller than the “common barn rat,” expresses the opinion that the white rats do not come from an accidental variation of the color of the coat of the common brown rat, but rather from a well-established variety of rat of unknown origin (Rodwell, 1858).

It is possible that all these colonies of white rats originated from multiple independent albino mutations occurring in different places. However, considering the low frequency of naturally occurring albino mutations and the high frequency of sightings of albino rodents in the 19th century in comparison to the previous ages, it is also possible that, as hypothesized by various authors of the time (such as Baikie, Heddle, Moss and Rodwell), some of these urban colonies of albino rodents were generated from the involuntary importation of individuals through ships from other countries where the albino variant was an already established breed, or from the escape or abandonment of domesticated albino rodents.

Indeed, the phenomenon of the abandonment of pet rodents in urban areas still exists even in the present day. For instance, Díaz-Berciano and Gallego-Agundez analyzed the records of a network of foster homes for abandoned rodents and rabbits in Madrid for the period from 2008 to 2021 (Díaz-Berciano and Gallego-Agundez, 2024). Over this period, 1,024 abandoned domestic rodents and rabbits were found. Of these, 749 (53.6%) were rodents and 475 (46.4%) were rabbits. Among the rodents, 202 (27.0%) were hamsters, 157 (21.0%) guinea pigs, 59 (7.9%) rats, 55 (7.3%) gerbils, 49 (6.5%) mice and 27 (3.6%) chinchillas. On the total number of abandoned rodents and rabbits, 1,009 animals (98.5%) were assigned to a foster house for adoption and 686 (67.0%) were subsequently given in adoption.

## 5 Conclusions: the historical significance of Moss’s behavioral study for scientific research and for society

Overall, the interspecies bonding case described by Samuel Moss is remarkable under several points of view. First, it is not only a case of social bonding between two animals of different species, but, most surprisingly, between a wild-caught rat and a dog trained to kill rats. Second, the case reported by Moss features an albino rat, a variety which at present is common, both as domestic pet and as laboratory animal, but at that time was extremely rare. Third, Moss presented a radically innovative image of the rat, with high historical significance for both science and general society.

In science, rodents were used as animal subjects for research studies since the 1650s, when the Irish-English chemist Robert Boyle (1627–1691) employed mice to investigate air respiration (Boyle, 1660). However, for more than a century and a half, exclusively physiological studies were performed. Moss’s study was the first rodent behavioral study. Remarkably, Moss described the rat’s behavior as sufficiently complex to be of interest for scientific investigation, in contrast to the view that rodents are “lower” animals that can be used only for physiological studies. Based on his behavioral observations, Moss presented the rat subject as capable of high cognitive functions and emotions, and hence worth of future behavioral investigation. This represented the first step for the employment of rodents as animal models in behavioral studies.

Moreover, Moss’s study represents an early example of awareness of the importance of the animal welfare of the studied subjects in scientific research. Indeed, the previous physiological studies on rodents, although performed by titans of the history of science, were very immature from the animal welfare point of view. In fact, the attention to the welfare of the rodents was very scarce or null. Most experiments were lethal and/or featured high levels of suffering. For instance, the aforementioned Boyle performed suffocation tests on mice to understand the maximal possible survival without air to breathe (Boyle, 1660). The Italian biologist Francesco Redi (1626–1697) carried out terminal starvation tests on domestic and field mice to assess the maximal time of survival without food (Redi, 1684). The Italian naturalist Felice Fontana (1730–1805) performed toxicological tests on guinea pigs evaluating the effects of inflammable air (Fontana, 1779), curare venom (Fontana, 1780, 1781, 1787), cherry-laurel poison (Fontana, 1781, 1787) and viper venom (Fontana, 1781, 1787). Moss, on the other hand, considered the individual value of the studied subject, which he regarded as an animal to take care of. From the 1780s, several authors in the United Kingdom advocated the recognition of animal rights and the importance of the defense of animal welfare, starting with the British philosopher Jeremy Bentham (1748–1832), who compared specist prejudices to racist prejudices (Bentham, 1789). For a history of animal welfare legislations and literature between the 17th and the 19th century, see section 5.1 of d'Isa and Abramson (2025). As a thermometer of the times, in 1824, just two years after that Moss had adopted Scugg and started his behavioral study, the politicians Richard Martin (1754–1834) and William Wilberforce (1759–1833), together with the clergyman Arthur Broome (1779–1837), established the Society for the Prevention of Cruelty to Animals (SPCA), the first animal welfare organization in the world (Moss, 1961). Moss was part of this change and practically implemented animal welfare principles in his study of rodent behavior.

The attention of Moss for the welfare of the studied subject had also implications for the scientific research methodology employed. Indeed, within behavioral science, Moss can be considered a pioneer of the ethological approach, which focuses on the long-term study of spontaneous behaviors, performed by the animals in their living context and possibly in a social environment. Such an approach is in opposition to a more artificial paradigm focusing instead on behaviors acutely elicited by the presentation of a stimulus as a consequence of previous reinforcement by the experimenter, assessed in a short session, in a context extraneous to the living environment of the animal and generally in absence of the social companions of the animal. Interestingly, the ethological approach of Moss was followed by many rodent behavioral scientists of the second half of the 19th century, who performed ethological studies on rodents in controlled settings (for a list of the studies, see d'Isa et al., 2024a).

For instance, from October 1869 to December 1870, the American zoologist and geologist Benjamin Cutler Jillson (1830–1899), professor at the Western University of Pennsylvania in Pittsburgh, studied the habits of two prairie dogs, at first in a large room of his university and subsequently in a pen outside his house (Jillson, 1871). Jillson relays that these rodents quickly developed a connection with him: “They soon became very tame, coming when called, and eating from my hand.” Moreover, Jillson describes the couple of prairie dogs as extremely united: “They were very affectionate, seldom quarrelling, and often standing with their fore paws on each other’s shoulders, rubbing their noses together.” One day, one of them had crawled in a hole of their room and was wandering about in the attic of the university building. Jillson recounts: “As often as I called, it would answer, and at length discovering that it had found its way to the ceiling, I removed a board from the floor of the room above and releasing it, returned it to its companion. The demonstrations of affection which followed would put to shame many a couple of higher intelligence.”

In 1873, the American naturalist George Henry Perkins (1844–1933), professor of natural history at the University of Vermont in Burlington, published the first study on the behavior of captive squirrels in a controlled setting (Perkins, 1873). Notably, Perkins studied the behavior of a couple of flying squirrels in their home-cage, which was endowed with a nest (a hemispheric wire netting filled with cotton and tow) and a running wheel for voluntary physical exercise. This was also the first scientific study on rodent wheel running.

In 1879, the American agricultural scientist Franklin Hiram King (1848–1911) studied the home-cage behavior of three flying squirrels, observing their circadian rhythms, play behavior and wheel-running (King, 1883). Additionally, King studied the climbing, diving and gliding behavior of the squirrels in their room when they were kept outside the cage. By leaving in the room a selection of acorns, hazel-nuts, hickory-nuts, pecans and English walnuts, King also observed the collecting and food-hoarding behavior of the flying squirrel, evaluating the specific food preferences for collection.

In 1894, the aforementioned Colin Campbell Stewart, from Clark University, created a system for the automated recording of the daily activity of rodents in their home-cage, which he employed with mice, squirrels and rats (Stewart, 1894). Stewart used this system to evaluate the effects of alcohol, barometric pressure and type of diet on voluntary motor activity (Stewart, 1894, 1898). Notably, Stewart’s 1894 machine is the first automated home-cage behavioral monitoring system in history.

Moss’s ethological approach of studying spontaneous behaviors continued to be employed also in the 20th century. While in the first half of the century, mainly under the influence of behaviorism, rodent behavioral testing featured mostly painful punishments or rewards based on previous states of deprivation (such as starvation or water-deprivation), in the second half of the century many behavioral tests based on spontaneous behavior were designed. For example, Boissier and Simon’s 16-hole-board (Boissier and Simon, 1962) or File and Wardill’s 4-hole-board (File and Wardill, 1975; d'Isa et al., 2021a) rely on the natural curiosity of mice for holes to make them look inside the holes of the board so that that their spatial memory can subsequently be assessed. In the object recognition test by Ennaceur and Delacour, mice are exposed to different objects, which are spontaneously explored based on their degree of novelty, so that recognition memory can be quantified (Ennaceur and Delacour, 1988; d'Isa et al., 2014). In the object location test, no object is changed across sessions, but the displacement of an object is employed to create a source of novelty and consequently generate greater levels of spontaneous exploration in the mice that remember the previous location (Ennaceur et al., 1997). The spontaneous alternation T-maze, originally experimented between the 1920s and the 1930s but more successfully launched from the 1950s, relies on the natural tendency of rodents to prefer exploring a novel arm of the maze over a familiar one (d'Isa et al., 2021b), as well as Gerlai’s more recent continuous alternation Y-maze (Gerlai, 1998).

At the beginning of the 21st century, there has been a revival of home-cage testing, starting from the creation of the automated home-cage behavioral monitoring system IntelliCage by the group of Hans-Peter Lipp and David Wolfer at the University of Zurich (Galsworthy et al., 2005; Lipp, 2005; Lipp et al., 2005; Kiryk et al., 2020; Lipp et al., 2024). Many other similar systems followed (Mingrone et al., 2020; Voikar and Gaburro, 2020; Kahnau et al., 2023), such as PhenoMaster (Urbach et al., 2008; König et al., 2020), Actual-HCA (Bains et al., 2016; Mitchell et al., 2020) and SmartKage (Ho et al., 2023). Remarkably, these systems, following an ethological approach, allow the continuous and long-term study of the spontaneous behavior of rodents in their living environment and with their social companions, without the interference of human handling or exposure to unfamiliar testing contexts (Mingrone et al., 2020; Voikar and Gaburro, 2020; Grieco et al., 2021; Kahnau et al., 2023). From the mid-20th century to present, there has been a continuously growing interest for animal welfare in biomedical sciences, as revealed by the biomedical archive PubMed, which shows a particularly sharp increase in the annual number of publications featuring the phrase “animal welfare” from the 1970s to present (d'Isa et al., 2024a). Currently, animal-friendly behavioral testing is considered a priority in rodent behavioral science and automated home-cage behavioral monitoring systems are a cutting-edge tool for animal-friendly behavioral testing (d'Isa and Gerlai, 2023). Interestingly, this ethological and welfare-optimizing approach has been experimented also in free-living rodents by deploying in the habitat of the species freely accessible testing chambers equipped with infrared video cameras, which could be freely visited and exited by the animals at any moment of the day (Stryjek et al., 2021, 2024; Parsons et al., 2023a, 2023b; d'Isa et al., 2024b).

Importantly, Moss changed the image of the rat not only for science, but also for the general society. Indeed, Moss surprisingly provided to the society an early example of keeping a tame rat as a pet, which at that time was still uncommon. Before Moss, rats were mainly considered as “vermin” and all the most famous guidebooks on pest control were putting the rats in first position in the list of the animals to exterminate (W. W., 1680; Smith, 1768; Simpson, 1772; Holland, 1782; Blencowe, 1812). Even in the mid-19th century, Rodwell’s handbook on the rat was entitled: *The Rat: Its History & Destructive Character* (Rodwell, 1858). The story of Moss’s tame rat generated a great sensation and was reported in several newspapers and books of the 19th century (for example: Cheltenham Chronicle, 1822; The Minerva, 1822; Stamford Mercury, 1823; Loudon, 1840; Wood, 1864; The Boy’s Friend, 1866; Pen and Plow, 1879; Chambers's Journal of Popular Literature, Science, and Art, 1878; Youmans and Youmans, 1878; Bidwell, 1879; The Australian Journal, 1879; Vincent, 1898), contributing to the change of image of rats and mice from obnoxious pest or vermin to little furry friends.

In 1880, the British journalist and writer Mary Anne Broome^22^ (1831–1911), published a collection of short stories for children, illustrated by the artist William John Hennessy (1839–1917), which included a story entitled *The White Rat*, that begins as follows: “I have often been asked which of all my pets I liked best, and I really think the white rat was the nicest and most amusing pet I ever had” (Barker, 1880). This short story appears as first and gives the name to the collection: *The White Rat and Some Other Stories*.

At the beginning of the new century, the British writer, illustrator and naturalist Beatrix Potter (1866–1943) wrote and illustrated extremely popular stories for children that featured anthropomorphized mice and rats, such as *The Tailor of Gloucester* (Potter, 1902), *The Tale of Two Bad Mice* (Potter, 1904), *The Roly-Poly Pudding* (Potter, 1908) and *The Tale of Mrs. Tittlemouse* (Potter, 1910).

In 1928, the American animators Walt Disney (1901–1966) and Ub Iwerks (1901–1971) created the character of Mickey Mouse, who starred in a series of animated cartoons, becoming the mascot of the Walt Disney Company and a worldwide pop culture icon (Kenworthy, 2001).

In 1945, the American novelist Elwyn Brooks White (1899–1985) released *Stuart Little*, a novel illustrated by the artist Garth Montgomery Williams (1912–1996) and focused on the story of the anthropomorphic mouse Stuart and his adventures in a human-sized world (White, 1945), which became a great classic of children literature.

In 1971, Robert Leslie Carroll Conly (1918–1973), better known by his pen name Robert C. O’Brien, an American novelist and journalist for the magazine *National Geographic*, published *Mrs. Frisby and the Rats of NIMH*, centered on a group of rats living in a colony, NIMH, with a literate and technologically society similar to the one of humans (O’Brien, 1971). In 1982 the book was adapted into an animated movie, *The Secret of NIMH*, by Don Bluth Productions.

More recently, in our century, Pixar Animation Studios produced the animated movie *Ratatouille*, which tells the story of a young rat, Remy, that refuses to eat garbage and dreams of becoming a French chef (Brandes and Anderson, 2011). Released in 2007, *Ratatouille* became a global blockbuster, winning the Oscar for Best Animated Feature in 2008 and igniting a craze for pet rats (Brayfield, 2010; O’Sullivan et al., 2014).

The case of Sanuel Moss was the first to impact the media with the idea of tame friendly rats, laying the ground for the change of the public opinion on rats that followed. In the end, the case described by Moss is not only an instance of interspecies bonding between rat and dog, but also between rat and human. Indeed, in his article (Moss, 1836) Moss does not only describe a scientific case, but also pays an homage to and remembers with pleasure a small animal that, even so many years after its death, Moss still defines “my little friend.”

## Footnotes

^1^Pittville is a residential suburb of Cheltenham built by the lawyer and real estate developer Joseph Pitt (1759–1842), from which the suburb receives its name. According to *Griffith’s New Historical Description of Cheltenham and Its Vicinity*, the buildings on Pitt’s land (hence also the kennel and stables used by Colonel Berkeley) were demolished in summer 1824, to leave space for the construction of the Pittville estate (Griffith, 1826). The “pleasure-ground” mentioned by Moss was a recreational area, within Pittville, that included a park with walks and rides (Pittville Park) and a monumental neoclassical spa building, known as the Pump Room, where a mineral water renowned for its beneficial properties on health was extracted from an underground source and offered to drink. The foundation stone of the Pump Room was laid on 4 May 1825 and the Pump Room was completed after 5 years of building, opening on 20 July 1830 (Rowbotham and Waller, 2004). Pittville Park, originally accessible only to the subscribers to the spa and to fee-paying non-subscribers, was made accessible to the public free of charge in 1954 (Waller et al., 2022). Today, Pittville Park and the Pittville Pump Room can still be visited and are among the major tourist attractions of Cheltenham.

^2^From here on, since the animal has received a proper name, we will use the pronouns “he” and “him” to refer to the rat, instead of “it.”

^3^Samuel Moss married Mary Jeffreys (1807–1867) on 24 February 1825 (Worcester Journal, 1825). Moss informs us that Scugg died 3–4 weeks after his marriage, meaning in late March (Moss, 1836).

^4^Translated from French into English by Raffaele d’Isa. The original French text of the quotation is reported in the Supplementary document.

^5^See footnote 4.

^6^Thomas Coryat (c. 1577–1617) was a popular English traveler and travel writer who, in 1608, undertook a five-month journey across Europe visiting France, Italy, Switzerland, Germany and the Netherlands. Three years after his return in England, Coryat published his memoirs of the journey, in which he provides a vivid description and comment, often with an eccentric style, of the habits of the people of the places he visited (Coryat, 1611). By choosing the pseudonym “Coriat Junior,” Paterson was proposing himself as a second Coryat.

^7^Although the front page of volume I bears the year 1767, it was actually published in 1768. Indeed, volume I contains a reference to *A Sentimental Journey Through France and Italy* by the British writer Laurence Sterne (1713–1768), which was first published in 1768. Moreover, the first review of volume I of Paterson’s work correctly reports 1768 as the book’s date of publication (The Monthly Review, 1768).

^8^The letter reports only the day and the month (“April 30”). The year, however, can be easily understood from an historical event mentioned in letter XXIX, written in the following June. In this letter, Edward relays that a friend of his, Tom Vernon, a boy aged 13, had just lost a leg in a naval battle in the Mediterranean Sea against the French ship Le Tonnant, after having being embarked on the British war ship The Tremendous as a midshipman under his uncle, who was the captain (Aikin, 1811). This fight between The Tremendous and Le Tonnant is part of the naval war between Great Britain and France which raged in the Mediterranean from May 1798 to August 1798 and culminated in the Battle of the Nile, which ended with the victory of the British fleet on 3 August 1798 (Willis, 2019).

^9^The Tower of London, which hosted the Royal Menagerie, was one of the most popular attractions in the city. As reported by a London guide from 1786, the Tower was open to the public and the entrance fee to visit it (including “the King’s menagerie for wild beasts,” the crown and regalia, the armory and the artillery) was, for all the sections together, 3 shillings and 10 pence for one person, and 2 shillings and 10 pence each for two persons (Trusler, 1786). The Tower’s menagerie was not only a popular touristic attraction, but also a source of inspiration for important artists of the time. For instance, it is most likely at the Tower of London that the Romantic poet William Blake (1757–1827) saw the tiger that inspired him to write his famous poem “The Tyger” (Miner, 1962), first published in 1794 (Blake, 1794).

^10^The Exeter Exchange, often written Exeter ‘Change, was a building in London’s major street The Strand, which hosted, in its upper floors, an animal menagerie of exotic animals that was active from 1773 to 1829, when the building was demolished (Talairach, 2021).

^11^The Cork Models were a long-popular exhibition of cork models reproducing architectures from classical antiquity, such as the Colosseum, set up in London by the French artist Richard Du Bourg (1738–1826). This exposition of cork models, formally known as “Classical Exhibition” was started by Du Bourg in 1775 and was closed in 1785 after a fire destroyed many of the models (Gillespie, 2017a, 2017b; Arnott-Davies, 2018). Du Bourg re-opened the exhibition in 1798 (Altick, 1978; Gillespie, 2017a) and kept it open until 1819, when he retired and sold his collection at an auction (Gillespie, 2017b; Arnott-Davies, 2018).

^12^Maillardet’s Automatic Exhibition was an exhibition of automata organized in London by the Swiss mechanician Henri Maillardet (1745–1830), who produced clocks, animal automatons and humanoid automatons (Edgeworth, 1801; La Belle Assemblée, 1807; Feltham, 1807). One of Maillardet’s most famous automata is the Draughtsman-Writer, a humanoid robot capable of drawing and writing poems, built in London in c. 1795 and currently part of the collection of the Franklin Institute in Philadelphia (Baron, 2018, 2019).

^13^Stephen Polito (1763/4–1814) was an Italian (birth name: Stefano Polito) menagerie businessman born in Moltrasio, a small town on Lake Como, in the Duchy of Milan (Grigson, 2016). From at least the 1780s, Polito toured England with travelling circuses. In 1810 Polito bought the static menagerie of Exeter Exchange in London and renamed it the Royal Menagerie. Polito combined the business of Exeter Exchange with a travelling menagerie that toured the United Kingdom in the summer months under the name Polito’s Menagerie. After Polito’s death in 1814, Exeter Exchange passed to his brother John Polito and in the same year it was bought by Edward Cross (1774–1754), a former employee of Stephen and John’s father-in-law, who renamed it Royal Grand National Menagerie. On the other hand, the travelling menagerie founded by Stephen Polito, even after his death, continued to tour under the name Polito’s Menagerie until the 1830s.

^14^Rodwell states that Ostin imported the white rats from France “about three years ago” and in the previous page he explicitly specifies the current year: “the present time (1851).”

^15^We cannot know the exact year of the interview. Nevertheless, it was likely around 1856. The interview with another rat-breeder in the same volume is dated 1856 and, in another section of the same volume, we can read “this year (1856)” (see Note 17).

^16^V. R., standing for Victoria Regina, is the royal cypher of Queen Victoria.

^17^In the interview, the man refers to the date 17 February 1856 as “the 17th of last February, 1856” (Mayhew, 1861). In another section of the book, we can read “this year (1856).”

^18^See footnote 4.

^19^Rats are born with their eyes closed and are blind during their first two weeks of life. Eye opening occurs generally at approximately 15–16 days after birth (Biel, 1940; Tan et al., 2015).

^20^The term “Analyst” stands for Chemical Analyst, the role held by John Horsley. In 1866 Horsley published a remarkable toxicology handbook which provided the description of a great number of poisons, with the methods to detect them, post-mortem or in the living (Horsley, 1866). In the front cover of the book, Horsley defines himself “Analytical Chemist.”

^21^See footnote 4.

^22^Formerly known as Lady Barker, Mary Anne became Lady Broome after marrying, in 1865, Sir Frederick Napier Broome (1842–1896), a colonial administrator in the British Empire, who was Governor of Mauritius from 1880 to 1883, Governor of Western Australia from 1883 to 1889 and Governor of Trinidad and Tobago from 1891 to 1897.
