# Supplementary material for: The first rodent behavioral study (1822) and the diffusion of human-bred albino rats and mice in the 19th century
Source: Front Psychol. 2025 Feb 3;15:1532975. doi: 10.3389/fpsyg.2024.1532975 (PMC11831927; doi:10.3389/fpsyg.2024.1532975)
Supplement: Supplementary file 1 [file Data_Sheet_1.pdf]

TRANSLATED QUOTATIONS

In the present supplementary document, we report all the original French texts of the quotations that in the article have been translated in English by Raffaele d'Isa.

Marie de Rabutin-Chantal (1626-1696), also known as Madame de Sévigné, on the pet white mouse of the philosopher François de La Rochefoucauld (de Rabutin-Chantal, 1818):

1. "M. de La Rochefoucauld vous mande qu'il a une souris blanche qui est aussi belle que vous; c'est la plus jolie bête du monde; elle est dans une cage" ("Monsieur de la Rochefoucauld tells you that he has a white mouse which is as beautiful as you; it's the prettiest beast we've ever seen; it's in a cage")

Louis Daniel Arnault de Nobleville (1701-1778) and François Salerne (1706-1760) on tame mice (de Nobleville and Salerne, 1757):

1. "Les Souris s'apprivoisent aisément; & quand une fois elles sont apprivoisées, elles ne cherchent point à s'échapper" ("Mice are easily tamed; and once they are tamed, they do not seek to escape")
2. "C'est un plaisir de voir avec quelle promptitude ces Animaux font tourner la roue: quand ils font las, ils rentrent dans la maisonnette pour s'y reposer. On peut accoutumer un Rat à la même manoeuvre" ("It is a pleasure to see how quickly these animals turn the wheel: when they get tired, they return to the house to rest. You can accustom a rat to the same maneuver")
3. "Nous avons vu une Souris blanche comme neige, que son Maître portoit par-tout avec lui dans une petite boëte; elle mangeoit familièrement du pain ou d'autre nourriture dans sa main, & après s'être promenée sur le bureau ou sur la table, elle rentroit d'elle-même dans sa boëte" ("We saw a mouse white as snow, which his master carried with him everywhere in a small box; it ate bread or other food with familiarity from his hand, and after walking around the desk or the table, it returned of its own will inside its box")

William Frédéric Edwards (1777-1842) on the breeding of albino mice by Jean-Antoine Colladon (Edwards, 1829):

1. "M. Coladon, pharmacien de Genève, pour multiplier les expériences sur les croisements de races et étendre nos idées sur ce sujet, éleva un grand nombre de souris blanches et de souris grises. Il en étudia attentivement les moeurs et trouva le moyen de les faire produire en les croisant. Il commença alors une longue suite d'expériences en accouplant toujours une souris grise à une souris blanche. Quel résultat attendez-vous? qu'il y ait eu souvent des mélanges? Non, jamais. Chaque individu des nouveaux produits était ou entièrement gris ou entièrement blanc, avec les autres caractères de la race pure; point de métis, point de bigarrure, rien d'intermédiaire" ("Monsieur Coladon, pharmacist from Geneva, to increase the experiences

on breed crossings and extend our ideas on this subject, raised a large number of white mice and grey mice. He carefully studied their habits and found a way to make them reproduce by crossings. He then began a long series of experiments by always mating a grey mouse with a white mouse. What result do you expect? That there were often mix-ups. No, never. Each individual of the new litter was either entirely grey or entirely white, with the other characteristics of the pure race; no mixed race, no variegation, nothing intermediate")

Auguste Henri André Duméril (1812-1870) on the colony of white rats of the Jardin des Plantes of the Muséum d'Histoire Naturelle of Paris (Duméril, 1854):

1. "Le rat blanc, qui se reproduit et s'élève très-facilement en captivité, nous fournira sans doute, dans quelques mois, une précieuse ressource. Depuis quatre semaines seulement, on a obtenu des produits assez nombreux des familles qu'on élève avec soin dans ce but, pour en donner aux Serpents qui, dans cette courte période, en ont pris douze avec avidité, ainsi que quinze rats rayés que depuis peu de temps aussi on nourrit dans des cages où leur reproduction paraît devoir être abondante comme celle des rats blancs" ("The white rat, which reproduces and raises very easily in captivity, will undoubtedly provide, in a few months, a valuable resource. Just from four weeks ago, we obtained a very numerous offspring from broods which we raise with care for this purpose, to give rats to the snakes that, in this short period, have eagerly taken twelve, as well as fifteen piebald rats that recently we have also been raising in cages where their reproduction seems to be abundant like that of the white rats")

References

de Nobleville, A. and Salerne, F. (1757). Suite de la matiere medicale de M. Geoffroy. Tome Sixiéme. Paris: Desaint & Saillant.

de Rabutin-Chantal, M. (1818). Lettres de Madame de Sévigné. Tome Troisième. Paris: J. J. Blaise.

Duméril, A. (1854). Notice historique sur la Ménagerie des Reptiles du Muséum d'Histoire Naturelle et observations qui y ont été recueilles. Paris: J. Claye et Compagnie.

Edwards, W. F. (1829). Des caractères physiologiques des races humaines considérés dans leur rapport avec l'histoire; Lettre a M. Amédée Thierry, auteur de l'Histoire des Gaulois. Paris: Compère Jeune.
